# Peel of Pomegranate Fruit (*Punica granatum*) Improves Glucose Homeostasis in Obese Mice: An Integrated In Vitro, In Vivo, and In Silico Molecular Docking Study

**DOI:** 10.3390/cimb48070670

**Published:** 2026-06-29

**Authors:** Prawej Ansari, Alexa D. Reberio, Asif Ali, Md Hamza Naquib, Sandeep Kumar, Dhivya C, Md Abeduzzaman Anon, Hajera Khatun, Md Ferdos Ahamed, Peter R. Flatt, Yasser H. A. Abdel-Wahab

**Affiliations:** 1 Centre for Diabetes Research, School of Biomedical Sciences, Ulster University, Coleraine BT52 1SA, UKpr.flatt@ulster.ac.uk (P.R.F.); y.abdel-wahab@ulster.ac.uk (Y.H.A.A.-W.); 2 Department of Pharmacology, National Medical College and Teaching Hospital, Birgunj 44300, Parsa, Nepal; 3 Department of Pharmacy, School of Pharmacy and Public Health, Independent University, Bangladesh (IUB), Dhaka 1229, Bangladesh; 4 Department of Pharmacy, Biomedicinskt Centrum BMC, Uppsala University, Husargatan 3, 75123 Uppsala, Sweden; 5 Department of Microbiology and Immunology, School of Medicine, Tulane University, New Orleans, LA 70112, USA; skumar7@tulane.edu

**Keywords:** diabetes, obesity, insulin, glucose, lipid, hyperglycemia, *Punica granatum*

## Abstract

Pomegranate (*Punica granatum*), a shrub belonging to the *Lythraceae* family, has long been recognized for its diverse pharmacological benefits, including potential roles in managing inflammation and diabetes. The present study explored the insulin-secretory and β-cell proliferative properties of the ethanol extract of *P. granatum* fruit peel (EEPG) and assessed its influence on glucose regulation in high-fat-fed diet-induced obese mice (HFDi-OM) through in vivo and in silico studies. In vitro, EEPG was found to activate cAMP-dependent pathways and regulate K_ATP_ channels, thereby enhancing glucose-stimulated insulin secretion from BRIN-BD11 β-cells, with partial reliance on extracellular calcium. EEPG promoted β-cell proliferation, as indicated by an increase in Ki-67 positive cells, and displayed inhibitory effects on glucose diffusion and starch hydrolysis, suggesting a capacity to delay carbohydrate digestion and absorption. Furthermore, EEPG demonstrated antioxidant activity by neutralizing free radicals. In an acute test, EEPG (at doses of 150 and 250 mg/5 mL/kg) improved oral glucose tolerance and elevated plasma insulin levels. Long-term oral treatment for 21 days to HFDi-OM led to a significant reduction in fasting blood glucose, body weight, and food and fluid intake. It also enhanced gastrointestinal motility and improved lipid profiles by increasing HDL and lowering total cholesterol, LDL, and triglycerides. The therapeutic properties of EEPG are likely attributed to its rich bioactive components, including flavonoids (quercetin, kaempferol, catechin, and epicatechin) and phenolic acids (ellagic acid), which exhibited strong multi-target binding affinities in in silico molecular docking studies toward SUR1, PDE4, PI3K, and α-amylase, thereby supporting enhanced insulin secretion, β-cell function and glucose homeostasis.

## 1. Introduction

Diabetes mellitus is a prevalent chronic metabolic condition that develops primarily from either inadequate insulin synthesis by the pancreas or impaired cellular responsiveness to insulin [1,2]. There has been a continuing rise in the diabetes population, and approximately 10.5% of adults worldwide have been diagnosed with diabetes. As of 2024, it is estimated that ~600 million people are currently suffering from diabetes, and that this number is expected to rise to ~900 million by 2050 [2,3,4]. There are two main forms of diabetes mellitus, which are both associated with various debilitating complications. Type 1 diabetes mellitus (T1DM) results from the autoimmune-mediated destruction of pancreatic β-cells, leading to minimal or absent insulin secretion. Type 2 diabetes mellitus (T2DM) is characterized by chronic hyperglycemia resulting from insulin resistance combined with progressive β-cell dysfunction [1,5]. Obesity, particularly visceral obesity characterized by excess fat accumulation around internal organs, is a major contributor to insulin resistance. To compensate for insulin resistance, pancreatic β-cells initially increase insulin secretion, resulting in hyperinsulinemia [6].

Pharmacological options for managing T2DM encompass several drug classes, including sulfonylureas, biguanides, sodium–glucose cotransporter-2 (SGLT2) inhibitors, dipeptidyl peptidase-4 (DPP-4) inhibitors, and incretin-based agents, glucagon-like peptide-1 (GLP-1) and glucose-dependent insulinotropic polypeptide (GIP) analogues. More recently, a dual GLP-1 and GIP receptor agonist, tirzepatide, has been introduced, which activates both receptors, and exerts superior metabolic control over blood glucose and associated weight loss compared to monotherapy with GLP-1 agonists [7,8]. However, these agents are expensive, in short supply and often come with a high rate of adverse events, such as heart failure, bladder cancer, gastrointestinal disorders (e.g., diarrhea, nausea, vomiting, constipation), and dermatological reactions such as skin rashes [8,9]. In addition to commonly used contemporary medications, people around the world, and especially in less affluent countries, are increasingly turning to alternatives to conventional medicine, including plant-based ancient remedies as a source of health benefits [10]. Natural sources, such as plants and their parts, are widely used, sparking renewed attention as complementary treatments rather than definitive treatments due to their better safety profile [10]. Plants and some natural sources contain bioactive compounds like polyphenols, terpenoids, saponins, carotenoids, alkaloids, and glycosides, all of which have been shown to have medicinal properties, whether used alone or in combination [5,11].

*Punica granatum*, a deciduous shrub belonging to the Lythraceae family, has been widely cultivated across Asia, Europe, the Middle East, and Mediterranean regions [1,5]. *Punica granatum* has been used in traditional medicine for centuries across Asia and the Middle East to cure a variety of diseases, including arthritis, inflammation and helminthiasis [12,13]. Previous studies demonstrated that pomegranate fruit peel extract exhibited significant glucose-lowering and lipid-lowering activities due to its antioxidant properties. These antioxidant properties also enhanced liver and renal function in hyperlipidemic mouse models [14]. Recent studies also indicate that pomegranate consumption can manage hyperglycemia and increase GLUT-4 gene expression, suggesting improved insulin sensitivity in obese T2DM patients [5,15]. Additionally, pomegranate juice can effectively lower oxidative stress in T2DM patients, potentially mitigating the early stages of diabetes-related oxidative damage [16].

The therapeutic effects of pomegranate fruit peel are attributed to its diverse array of bioactive compounds, including polyphenols, ellagitannins (e.g., pedunculagin, punicalagin, valoneic acid dilactone), gallic acid, ellagic acid, and anthocyanins [11,17]. Preclinical studies showed that ellagic acid, kaempferol, quercetin, epicatechin, and catechin exhibit promising hypoglycemic effects, also potentially improving insulin secretion, sensitivity, and glucose uptake [18,19,20]. Although several studies have reported the beneficial effects of *Punica granatum* fruit peel on oxidative stress, inflammation, glucose tolerance, lipid metabolism, and glycemic control, the underlying mechanisms, particularly those related to insulin secretion and β-cell function, remain inadequately understood. Therefore, this study employs an integrated in vitro, in vivo, and in silico approach to investigate the mechanisms by which pomegranate fruit peel improves glycemic control and preserves pancreatic β-cell function.

## 2. Materials and Methods

### 2.1. Collection and Preparation of Extracts

Ripe fruits of *Punica granatum* L. were obtained from the Karwan Bazar, Dhaka, Bangladesh, on 9 May 2022. The botanical identification was confirmed by an expert taxonomist at the National Herbarium of Bangladesh, where a voucher specimen was deposited under accession number DACB 135372. The extract was prepared using a standard method involving grinding of air-dried peel, 72 h extraction in 80% ethanol, filtration and subsequent freeze drying of the concentrated filtrate as described previously [21].

### 2.2. In Vitro Insulin-Releasing Studies

To examine the effect of ethanol extract of *Punica granatum* fruit peel (EEPG) on insulin release in vitro, clonal pancreatic BRIN-BD11 β-cells, developed through electrofusion of primary rat islets with RINm5F cells, were used. The origin, characteristics and culture of these cells have been described elsewhere [22]. Following short preincubation, the cells were exposed for 20 min at 37 °C to Krebs–Ringer Bicarbonate Buffer (KRBB) containing 5.6 mM glucose and varying concentrations of EEPG. To determine the underlying secretory mechanisms, the effects of EEPG were assessed with/without various insulin secretagogues and inhibitors. These included: IBMX (3-isobutyl-1-methylxanthine), tolbutamide, 30 mM KCl, 10 mM alanine, verapamil, and diazoxide. Insulin released from the cells was measured by an insulin radioimmunoassay [22,23]. Cytotoxicity was assessed via measurement of lactate dehydrogenase (LDH) leakage, employing the CytoTox 96 non-radioactive assay kit (Promega, Southampton, UK), following the manufacturer’s guidelines [24].

### 2.3. Proliferation In Vitro

BRIN-BD11 cells were treated with 40 and 200 µg/mL of EEPG to scrutinize its effect on β-cell proliferation. Cell proliferation was evaluated using Ki-67 immunostaining, and glucagon-like peptide-1 (GLP-1) was utilized as a reference drug. Cells were cultured on coverslips (40,000 cells per coverslip) in RPMI-1640 medium containing the respective treatments for 18 h at 37 °C in a CO_2_ incubator. The cells were first washed (PBS) and fixed (4% paraformaldehyde). Antigen retrieval was then performed, and non-specific binding was blocked using 1.1% bovine serum albumin (BSA). Ki-67 expression was detected using a primary antibody (1:500 dilution; Abcam, Cambridge, UK) followed by Alexa Fluor^®^ 488 secondary antibody (1:400 dilution; Invitrogen, Waltham, MA, USA). Nuclear staining was performed with DAPI (350 nm), and fluorescence imaging was carried out using an Olympus BX51 microscope (Olympus System, Tokyo, Japan) equipped with appropriate filters (FITC (488 nm) and TRITC (594 nm) and camera systems [24].

### 2.4. DPPH Radical Scavenging Activity Assay In Vitro

The antioxidant potential of EEPG was determined via the 2,2-diphenyl-1-picrylhydrazyl (DPPH) method. EEPG (1.6–5000 µg/mL) or L-ascorbic acid (reference standard) was mixed with 0.2 mmol/L DPPH solution and incubated for 30 min at room temperature in darkness. The number of replicates was three (*n* = 3). Absorbance of remaining DPPH radical was measured at 517 nm using a UV/VIS spectrophotometer (Spectronic 200, Mettler-Toledo, Hamilton, New Zealand) [25].

### 2.5. Starch Digestion In Vitro

The effect of EEPG on starch breakdown was studied using a solution of 100 mg starch in 50 mL water, with or without EEPG, or acarbose as a positive control (an established α-glucosidase inhibitor). Samples were treated with 40 µL of 0.01% α-amylase (from *Bacillus licheniformis*) at 80 °C for 20 min, and then the mixture was diluted to 10 mL, and a 1 mL aliquot was mixed with 2 mL of 0.1 M sodium acetate buffer (pH 4.75). Subsequently, 30 µL of 0.1% amyloglucosidase (from *Rhizopus* sp.) (Sigma-Aldrich, St. Louis, MO, USA) was added and incubated at 60 °C for 30 min. Glucose released was measured via the Glucose Oxidase-Phenol Aminophenazone (GOD-PAP) method (Randox GL 2623) and expressed as a percentage relative to control. Absorbance was taken at 500 nm against a reagent blank at 37 °C [26].

### 2.6. Glucose Diffusion In Vitro

The influence of EEPG on glucose movement was assessed using cellulose ester dialysis membranes (dimensions: 20 cm × 7.5 mm; molecular weight cut-off: 2000; Spectra/Por^®^, Spectrum, Amsterdam, The Netherlands). The glucose diffusion retardation capacity of EEPG was investigated in vitro by incubating varying concentrations of the extract with a 2 mL volume of 0.9% NaCl (BDH Chemicals Ltd., Poole, UK) supplemented with 220 mM glucose inside the dialysis tubing. Both ends of each membrane tube were tightly sealed and placed in a 50 mL centrifuge tube (Orange Scientific, Orange, CA, USA) containing 0.9% NaCl (45 mL). The tubes were put on an orbital shaker and maintained at 37 °C for 24 h, followed by GOD-PAP assay of the external solution [22,26].

### 2.7. Animal Studies

Male Swiss albino mice (20–25 g) were housed at the Animal House of Independent University, Bangladesh, under controlled environmental conditions (22 ± 3 °C, 55–65% relative humidity) with *ad libitum* access to food and water and a 12 h light/dark cycle. Following a one-week acclimation period, the mice were maintained either on a standard chow diet (normal control group) or a high-fat diet (HFD) for 6–8 weeks to induce obesity and impaired glucose regulation. A standard diet provided 12.99 kJ/g of energy, whereas the HFD contained powdered standard food (19.4%), sugar (17.5%), beef fat (21%), condensed milk (39.5%), vitamin B complex (1.1%), and salt (1.5%), which provided 26.15 kJ/g of energy. Mice with fasting blood glucose > 6.0 mmol/L and body weight of 40–45 g were selected as HFDi-OM for experiments [24]. Animals were assigned to the following groups (*n* = 6 per group):

Group 1: Lean control (saline, 5 mL/kg);

Group 2: HFDi-OM control (saline, 5 mL/kg);

Group 3: HFDi-OM + EEPG (150 mg/5 mL/kg);

Group 4: HFDi-OM + EEPG (250 mg/5 mL/kg);

Group 5: HFDi-OM + glibenclamide (5 mg/5 mL/kg).

To examine gastrointestinal (GI) motility, mice were first euthanized with a terminal dose of sodium pentobarbital (50 mg/5 mL/kg) administered intraperitoneally. A midline abdominal incision was then performed, extending from the pelvic area to approximately 1 cm below the xiphoid process.

### 2.8. Acute Oral Glucose Tolerance

After an overnight fast (12 h), normal mice and HFDi-OM were given glucose (2.5 g/5 mL/kg) by oral gavage, either alone or together with EEPG (150 or 250 mg/5 mL/kg) or glibenclamide (5 mg/5 mL/kg). Blood samples were collected from the tail vein at 0, 30, 60, 120, and 180 min for glucose measurement (Ascensia Contour meter, Bayer, Newbury, UK). Plasma insulin concentrations were determined using an Insulin ELISA kit on centrifuged samples, as previously described [22].

### 2.9. Chronic Studies

For 21 days, treatment groups received EEPG (150 and 250 mg/5 mL/kg) or glibenclamide (5 mg/5 mL/kg) twice daily, while control groups (Normal mice and HFDi-OM) received saline (5 mg/5 mL/kg). Fasting blood glucose, body weight, and food/water intake were assessed on a three-day interval, as previously described [23]. For statistical analysis of repeated measurements, two-way ANOVA followed by Bonferroni’s post hoc test for multiple-group comparisons was used.

### 2.10. Lipid Profile Test

At the end of chronic treatment, mice were anesthetized, and blood was collected via cardiac puncture into heparinized tubes. Plasma was separated by centrifugation (12,000 rpm, 5 min) and analyzed for HDL, total cholesterol (TC), triglycerides (TG), and LDL using colorimetric assay kits (Elabscience, Houston, TX, USA; Biolabo SAS, Maizy, France) with an automated analyzer as previously reported [27].

### 2.11. Gut Motility In Vivo

Following a 12 h fast, gastrointestinal motility was assessed by monitoring the transit distance of a barium sulfate (BaSO_4_) milk solution. EEPG (150 or 250 mg/5 mL/kg) or glibenclamide (5 mg/5 mL/kg) was administered orally 1 h prior to the BaSO_4_ solution, as previously reported [24]. Following a 15 min period post-administration of BaSO_4_, the mice were humanely euthanized, and the entire small intestine was excised. The BaSO_4_ transit distance was subsequently measured and expressed as a percentage of the total small intestinal length, measured from the pylorus to the ileocecal junction.

### 2.12. Phytochemical Screening

A preliminary phytochemical screening of EEPG was conducted to identify the presence or absence of alkaloids, tannins, flavonoids, terpenoids, steroids, saponins, glycosides, and reducing sugars, following modifications to the method previously described by Ansari et al. [24,28,29]. In brief,

- Alkaloids were detected by treating EEPG with Dragendorff’s solution and acidifying with hydrochloric acid (HCl); the presence of alkaloids was indicated by the emergence of an orange-red precipitate.
- Tannins were detected by mixing EEPG with a few drops of 10% lead acetate; the presence of tannins was confirmed by the formation of a white precipitate.
- To evaluate the presence of flavonoids, EEPG was mixed with methanol and heated, followed by the addition of magnesium metal and a few drops of HCl, resulting in a pink color.
- To identify the presence of phenolic acids, the EEPG peel extract was combined with distilled water, followed by a few drops of 10% ferric chloride. Formation of blue/green color indicates the presence of phenols.
- The presence of terpenoids was assessed by adding acetic anhydride and concentrated sulfuric acid to EEPG. The formation of blue/green rings indicated a positive result.
- Steroids were distinguished by combining EEPG with chloroform, and then adding acetic anhydride and concentrated sulfuric acid, which caused the formation of a bluish-green color.
- The presence of saponins was identified by combining EEPG with distilled water; the test tube was shaken vigorously, which resulted in the production of a stable foam.
- For glycoside testing, EEPG was mixed with a few drops of glacial acetic acid and ferric chloride. Concentrated sulfuric acid was then added. The appearance of a bluish-green color indicated the presence of glycosides.
- To determine reducing sugars, EEPG, and a few drops of Fehling’s reagent were combined. The mixture was then shaken and briefly heated, producing a red-brick precipitate.

### 2.13. Molecular Docking Analysis

Molecular docking is an effective process for exploring receptor–protein potential drug molecules for disease treatment. The three-dimensional molecular configuration of the protein was downloaded from the RCSB Protein Data Bank. Water molecules, co-crystal ligands, heteroatoms, and unexpected chains were deleted using the Discovery Studio Visualizer 2021 software to create the protein chain [30]. The 3-dimensional structures of SUR1 (PDB ID: 7WIT) [31], PDE4 (PDB ID: 3G4K) [32], PI3K (PDB ID: 4FLH) [33], and α-amylase (PDB ID: 1HNY) [34] were prepared, where Chain A has been selected for each structure, followed by the addition of polar hydrogens and Kollman united-atom charges using AutoDock Tools version 1.5.6. After protein preparation, all ligands (present in EEPG) were downloaded from the PubChem database with 3D-PDB structure. Avogadro software version 1.2.0 was used to optimize geometry. Using the Swiss-PdbViewer (Version 4.1.0), the protein chain was subjected to energy reduction via the conjugate gradient approach to eliminate poorly interacting protein atoms [35]. Molecular docking of the whole protein using PyRx (Python Prescription 0.8) was performed. The Auto-Dock Vina plugin in the PyRx tool was used to perform molecular docking of the target protein with key phytochemicals identified in EEPG and retrieved from PubChem, including quercetin (CID: 5280343), kaempferol (CID: 5280863), catechin (CID: 9064), epicatechin (CID: 72276), and ellagic acid (CID: 5281855) [30,31]. During the docking process, Gasteiger charges were added to ligands, the exhaustiveness value was set to 8, and the binding site was defined using maximum grid center coordinates in three dimensions for whole-protein docking. Blind docking was done to cover the whole protein, which allows unbiased identification of the preferred binding site [36]. The compounds were subsequently ranked according to their binding affinity scores (kcal/mol). Molecular interactions of the top-ranked protein–ligand complexes were visualized using BIOVIA Discovery Studio 2021, while the 3D structures of the protein–ligand complexes were generated using ChimeraX version 1.11.1 [32].

### 2.14. Statistical Analysis

Experimental data are expressed as mean ± SEM and were analyzed using GraphPad Prism 10. Statistical significance was set at *p* < 0.05. Comparisons between two groups were performed using the unpaired Student’s *t*-test. For multiple-group comparisons, one-way ANOVA, two-way ANOVA, or repeated-measures two-way ANOVA, as appropriate, followed by Bonferroni’s post hoc test, were used.

## 3. Results

### 3.1. Insulin Release with EEPG

**Figure 1:**
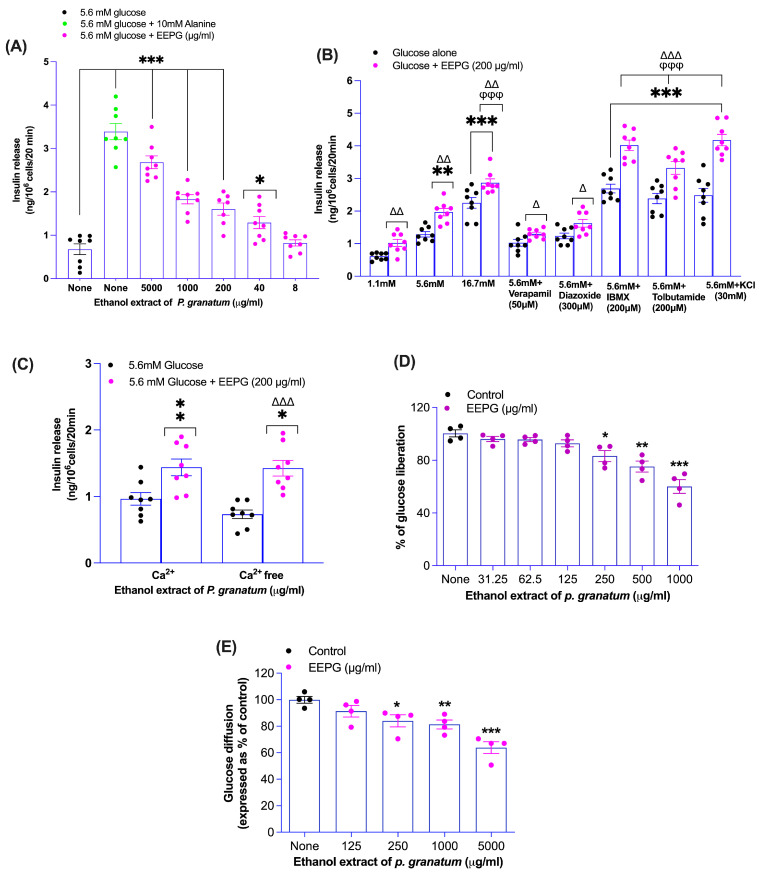
Effects of ethanol extract of *P. granatum* fruit peel (EEPG) on insulin secretion from clonal pancreatic BRIN BD11 β-cells, (**A**) concentration dependency, (**B**) with known modulators or inhibitors, (**C**) with or without extracellular calcium, (**D**) starch digestion, and (**E**) glucose diffusion. Dose-dependent insulin secretion, insulin secretion with or without modulators and extracellular calcium, and percentage of glucose liberated from in vitro starch digestion and glucose diffusion are represented in the scatter dot plot. Values are mean ± SEM; *n* = 8 for insulin secretion and *n* = 4 for starch digestion and glucose diffusion. * *p* < 0.05, ** *p* < 0.01, *** *p* < 0.001 compared to control (Figure A–C vs. 5.6 mM glucose alone). ^φφφ^ *p* < 0.001 compared to 5.6 mM glucose with EEPG (Figure B). ^Δ^ *p* < 0.05, ^ΔΔ^ *p* < 0.01, ^ΔΔΔ^ *p* < 0.001 compared to respective incubation without EEPG (Figure B,C). EEPG denotes ethanol extract of *P. granatum* fruit peel.

Within the 40 and 5000 µg/mL dosage range, EEPG in the presence of 5.6 mM glucose led to a dose-dependent increase (*p* < 0.05, *p* < 0.001; Figure 1A) in insulin secretion from BRIN-BD11 cells. Alanine (10 mM), used as insulin secretagogues, enhanced (*p* < 0.001; Figure 1A) insulin secretion to a level comparable with higher concentrations (5000 µg/mL) of EEPG. Concentrations up to 200 µg/mL showed no adverse impact on cell viability, while higher doses elevated LDH leakage (*p* < 0.05–0.01), indicating compromised cell membrane integrity and reduced viability.

### 3.2. Insulin Release with EEPG in Presence of Known Modulators and Absence of Extracellular Ca^2+^

Further research was carried out to better understand the mechanisms underlying the insulinotropic effects of non-cytotoxic doses of EEPG (200 µg/mL). The presence of insulin modulators, as illustrated in Figure 1B, such as high glucose (16.7 mM, *p* < 0.01), the non-selective phosphodiesterase inhibitor IBMX (200 μM, *p* < 0.001), and the sulfonylurea receptor 1 (SUR1) agonist tolbutamide (200 μM, *p* < 0.001), substantially enhanced insulin secretion induced by EEPG from BRIN BD11 β-cells. Tolbutamide showed its action via blocking SUR1 channels and inducing membrane depolarization, and enhanced the secretion of insulin. Insulin inhibitors, such as L-type voltage-gated calcium channel (VGCC) blocker verapamil (50 µM), K_ATP_ channel opener diazoxide (300 µM) and depletion of extracellular Ca^2+^, partially (*p* < 0.05; Figure 1B,C) attenuated EEPG-induced insulin secretion. EEPG sustained its ability to enhance insulin production in cells depolarized with 30 mM KCl (*p* < 0.001; Figure 1B), illustrating a mode of action in addition to membrane depolarization and triggering of calcium ion influx.

### 3.3. Proliferation of β-Cells In Vitro with EEPG

**Figure 2:**
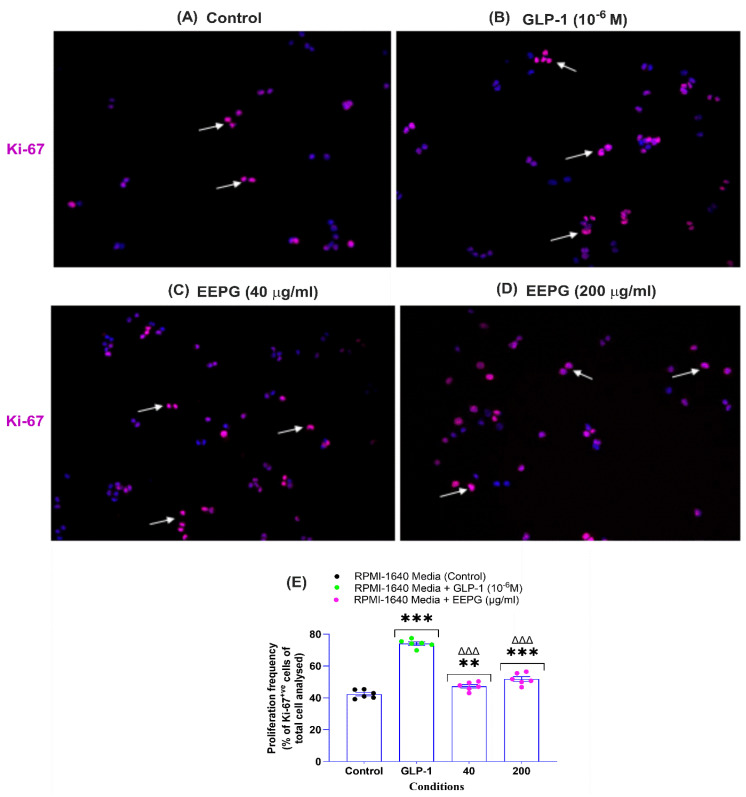
Effects of ethanol extract of *P. granatum* fruit peel (EEPG) on (**A**–**E**) BRIN-BD11 β-cell proliferation. BRIN-BD11 cells were cultured for 18 h with either EEPG (at 40 and 200 μg/mL) or GLP-1 (10^−6^ M), and their proliferation was assessed by Ki-67 staining. Representative images show cells stained for Ki-67 (pink) with DAPI (blue). Arrows indicate positively stained cells. (**A**–**D**) show proliferation images under four conditions: (**A**) control, (**B**) GLP-1 (10^−6^ M), (**C**) EEPG (40 μg/mL), and (**D**) EEPG (200 μg/mL). The percentage of Ki-67^+ve^ cells is represented in the scatter dot plot. All values are mean ± SEM with *n* = 6. ** *p* < 0.01 and *** *p* < 0.001 compared with RPMI-1640 medium alone. ^ΔΔΔ^ *p* < 0.001 compared with GLP-1 (10^−6^ M) alone.

Representative images of proliferating BRIN BD11 β-cells under three different conditions are shown in Figure 2A–D: (A) control, (B) GLP-1 (10^−6^ M), and (C, D) EEPG (at 40 and 200 μg/mL). In comparison to the control group, quantitative analysis (Figure 2E) indicated that EEPG substantially increased β-cells proliferation at concentrations of 40 μg/mL (*p* < 0.01) and 200 μg/mL (*p* < 0.001). GLP-1 (10^−6^ M), used as a reference drug, was more potent (*p* < 0.001; Figure 2E) compared to both doses of EEPG.

### 3.4. DPPH Scavenging Activity Assay In Vitro

In the DPPH assay, EEPG demonstrated concentration-dependent free radical scavenging activity, producing 9–91% inhibition across the tested range (1.6–5000 µg/mL). L-ascorbic acid, the reference compound, produced 11–96% inhibition at the same concentrations. Both agents showed statistically significant differences compared to the control (*p* < 0.05–0.001), as shown in Table 1.

### 3.5. Starch Digestion In Vitro with EEPG

EEPG significantly reduced glucose liberation during starch hydrolysis, with inhibition ranging from 10 to 30% (*p* < 0.05–0.001; Figure 1D) at concentrations of 250–1000 µg/mL. The reference compound acarbose (62.5–1000 mg/mL) also suppressed starch digestion (18–85%).

### 3.6. Glucose Diffusion In Vitro with EEPG

As shown in Figure 1E, EEPG considerably reduced glucose diffusion compared to control (*p* < 0.05–0.001). It reduced glucose diffusion by 15–35%, with the greatest inhibition (35%) at a 5000 µg/mL concentration.

### 3.7. Acute Oral Glucose Tolerance with EEPG

**Figure 3:**
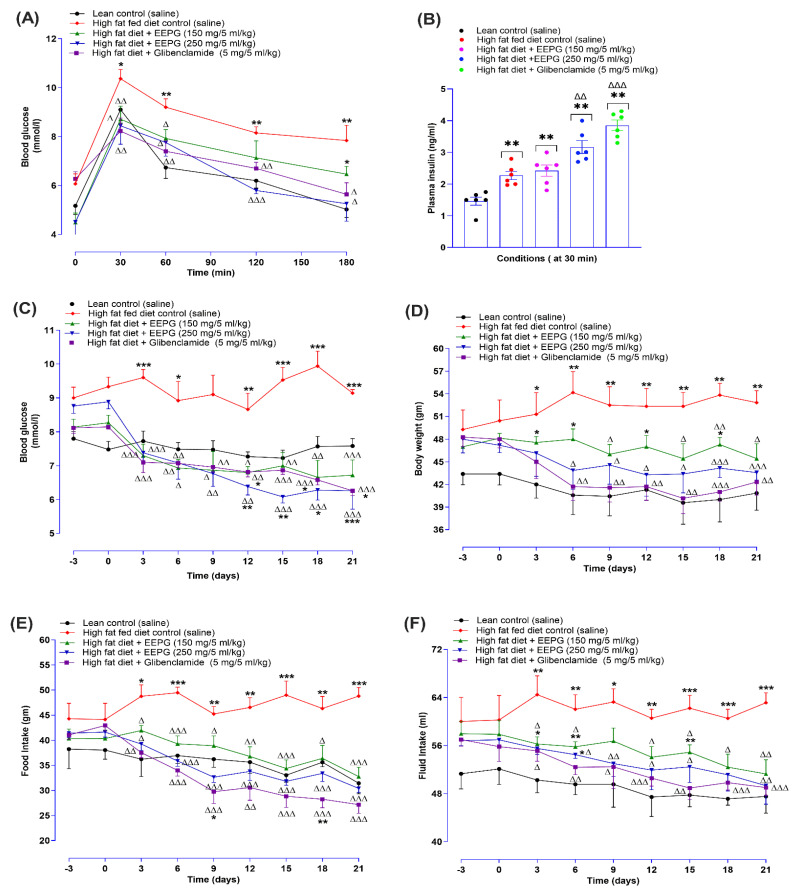
Effects of acute and chronic treatment (twice-daily) with ethanol extract of *P. granatum* fruit peel (EEPG) on (**A**) oral glucose tolerance and (**B**) plasma insulin level at 30 min, (**C**) body weight, (**D**) blood glucose, (**E**) food intake, and (**F**) fluid intake over 21 days of administration to high-fat-fed mice represented as line and bar graphs. Following an overnight fast, blood glucose was measured from the tail tips of high-fat-fed mice before and after they received an oral glucose challenge (2.5 mg/5 mL/kg body weight, control) with or without EEPG (150 and 250 mg/5 mL/kg body weight) or glibenclamide (5 mg/5 mL/kg). Subsequently, parameters were assessed at 3-day intervals before and after treatment. All values are presented as the mean ± SEM for *n* = 6. Statistical significance is indicated as follows: * *p* < 0.05, ** *p* < 0.01, *** *p* < 0.001 compared to the control (saline) group; ^Δ^ *p* < 0.05, ^ΔΔ^ *p* < 0.01, ^ΔΔΔ^ *p* < 0.001 compared to the high-fat-fed diet control mice. Glibenclamide served as a positive control.

Oral administration of EEPG (250 mg/5 mL/kg) in HFDi-OM significantly improved (*p* < 0.05–0.001; Figure 3A) glucose tolerance compared to control at all measured time points. At the lower dose (150 mg/5 mL/kg), significant (*p* < 0.05; Figure 3A) improvement was evident only at 30 and 60 min. Glibenclamide (5 mg/5 mL/kg) consistently reduced glycemia throughout the test. Plasma insulin levels were also elevated (*p* < 0.01–0.001; Figure 3B) in EEPG-treated mice (both doses) at 30 min post-glucose challenge.

### 3.8. Chronic Study with EEPG

In a 21-day study, oral gavage of EEPG (150 and 250 mg/5 mL/kg) and glibenclamide (5 mg/5 mL/kg) twice daily substantially decreased fasting blood glucose (FBG) levels in HFDi-OM, as depicted in Figure 3C. Both EEPG doses (150 and 250 mg/5 mL/kg) significantly (*p* < 0.05–0.001) reduced FBG levels from day 3 onwards. Notably, the higher dose (250 mg/5 mL/kg) led to a more sustained decrease in FBG throughout the 21-day study. The standard drug, glibenclamide (5 mg/5 mL/kg), also consistently reduced (*p* < 0.05–0.001; Figure 3C) FBG levels throughout the experimental period.

Figure 3D illustrates the corresponding long-term effects on body weight in HFDi-OM. Compared to the HFDi-OM control group, EEPG at 150 mg/5 mL/kg decreased (*p* < 0.05–0.01) body weight from day 9 onwards. Furthermore, the higher dose of 250 mg/5 mL/kg EEPG and glibenclamide (5 mg/5 mL/kg) gradually decreased (*p* < 0.05–0.001) body weight, with a consistent effect observed from day 6 onwards.

Oral gavage of EEPG (150 and 250 mg/5 mL/kg) for 21 days significantly (*p* < 0.05–0.001; Figure 3E,F) decreased food and fluid consumption from day 3 onwards. Similarly, the established drug, glibenclamide (5 mg/5 mL/kg), also significantly reduced (*p* < 0.01–0.001; Figure 3E,F) food and fluid consumption consistently in HFDi-OM.

### 3.9. Lipid Profiling with EEPG

**Figure 4:**
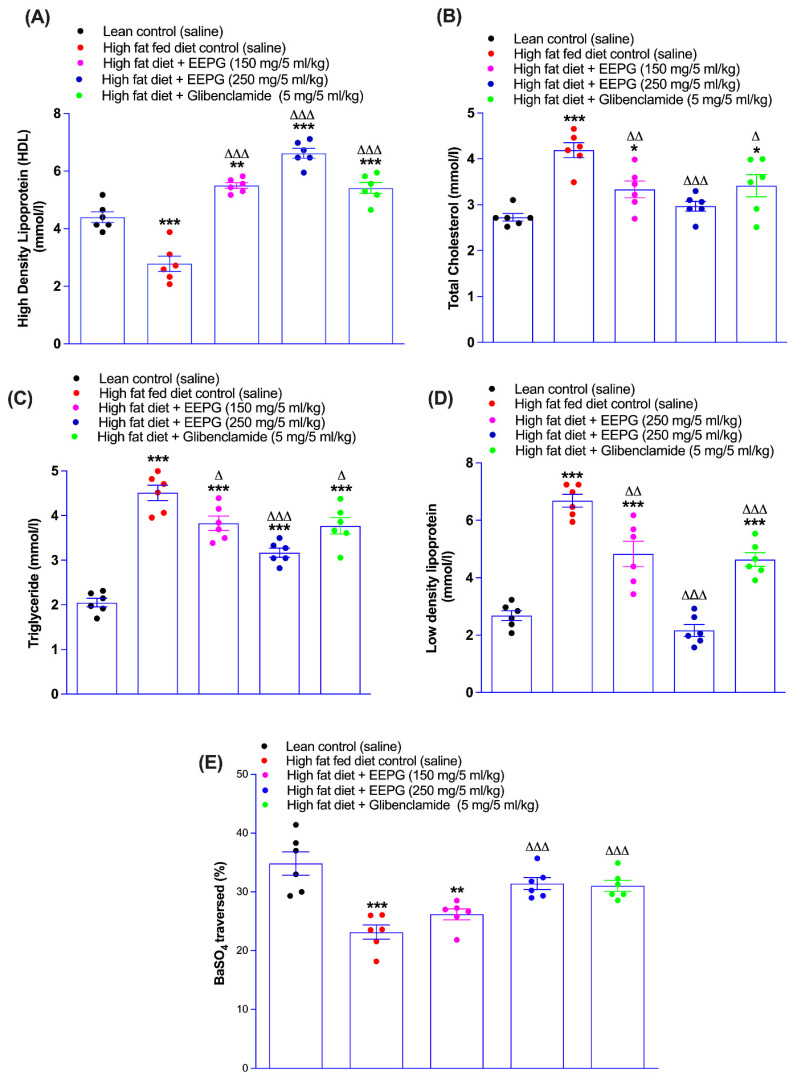
Effects of 21-day (twice-daily) oral administration of ethanol extract of *P. granatum* fruit peel (EEPG) on (**A**) HDL cholesterol, (**B**) total cholesterol, (**C**) triglyceride and (**D**) LDL cholesterol, and (**E**) gut motility in lean mice and high-fat-fed mice expressed as scatter dot plots. Following 21 days of treatment with twice-daily administration of either EEPG (at 150 and 250 mg/5 mL/kg body weight) or glibenclamide (5 mg/5 mL/kg body weight), lipid profile parameters were measured. Gut motility was assessed in 12 h starved mice by measuring BaSO_4_ transit length after BaSO_4_ solution ingestion. All values are expressed as mean ± SEM (*n* = 6 mice). Statistical significance is indicated as * *p* < 0.05, ** *p* < 0.01, *** *p* < 0.001 compared to the control (saline) group, and ^Δ^ *p* < 0.05, ^ΔΔ^ *p* < 0.01, ^ΔΔΔ^ *p* < 0.001 compared to the high-fat-fed diet control (saline) mice. Glibenclamide served as a positive control.

After 21 days, both doses of EEPG (150 and 250 mg/5 mL/kg) or glibenclamide (5 mg/5 mL/kg) improved (*p* < 0.05–0.001; Figure 4A–D) lipid profiles in HFDi-OM. Treatment elevated HDL cholesterol (*p* < 0.001; Figure 4A) and reduced total cholesterol, triglycerides, and LDL cholesterol (*p* < 0.05–0.001; Figure 4B–D). The higher dose (250 mg/5 mL/kg) exhibited greater potency compared with the lower dose and even outperformed glibenclamide in some parameters.

### 3.10. Gut Motility with EEPG

Figure 4E represents the effects of EEPG on gut motility after 21 days of administration. No significant change was observed with a 150 mg/5 mL/kg dose of EEPG. However, the higher dose (250 mg/5 mL/kg) substantially (*p* < 0.001; Figure 4E) promoted gut motility. Moreover, gut motility was also significantly (*p* < 0.001; Figure 4E) increased by glibenclamide at 5 mg/5 mL/kg.

### 3.11. Phytochemical Screening with EEPG

Qualitative analysis of EEPG was conducted to identify its anticipated phytoconstituents. The screening revealed that EEPG is rich in flavonoids, phenols, terpenoids, steroids, saponins and reducing sugars (Table 2). Previous studies have reported that ethanolic extracts of pomegranate peel contain flavonoids, such as quercetin, kaempferol, catechin, and epicatechin. These extracts are also rich in phenolic acids, including ellagic acid and gallic acid (Table 2) [37].

### 3.12. Molecular Docking with Phytochemicals

**Figure 5:**
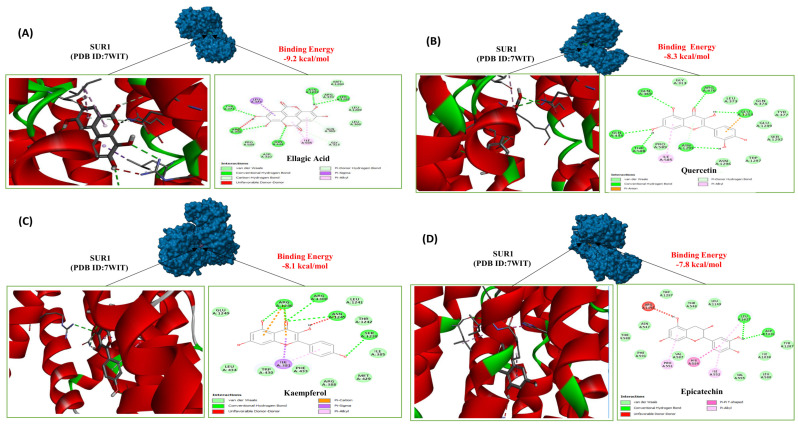
Molecular docking interaction of selected phytochemicals: (**A**) ellagic acid, (**B**) quercetin, (**C**) kaempferol, and (**D**) epicatechin derived from ethanol extract of *P. granatum* fruit peel (EEPG) with receptor SUR1 (PDB ID:7WIT). Docking analysis demonstrated favourable binding affinities of the phytochemicals toward the SUR1 active site. Ellagic acid exhibited the strongest binding affinity (−9.2 kcal/mol), forming conventional hydrogen bonds with SER A:1292 and ASN A:1293. Quercetin and kaempferol showed binding energies of −8.3 and −8.1 kcal/mol, respectively, with quercetin forming hydrogen bonds with ASN A: 1293, GLU A: 1253, THR A: 588, and π–alkyl interactions with ILE A: 585 within the binding pocket. These interactions suggest the potential modulation of SUR1-associated ATP-sensitive potassium channels, supporting the insulinotropic and glucose homeostasis effects of EEPG.

**Figure 6:**
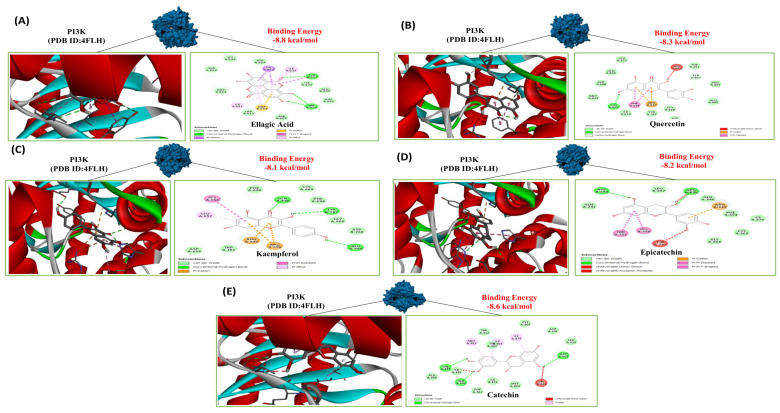
Molecular docking interaction of selected phytochemicals: (**A**) ellagic acid, (**B**) quercetin, (**C**) kaempferol, (**D**) epicatechin, and (**E**) catechin derived from ethanol extract of *P. granatum* fruit peel (EEPG) with receptor PI3K (PDB ID:4FLH). Docking analysis revealed strong binding affinities of five *Punica granatum*-derived phytochemicals toward the PI3K active site. Among the compounds, ellagic acid exhibited the highest binding affinity (−8.8 kcal/mol), showing several interactions, such as π–sulfur interaction with MET A:953, π–sigma interaction with ILE A:963, and π–alkyl interactions with ILE A:881, ILE A:963, and ILE A:831. Quercetin and catechin showed binding energies of −8.3 kcal/mol and −8.6 kcal/mol, respectively, while epicatechin and kaempferol exhibited binding energies of −8.2 and −8.1 kcal/mol. These findings suggest that these phytochemicals, particularly ellagic acid, may promote β-cell proliferation through activation of the PI3K/Akt signaling pathway.

**Figure 7:**
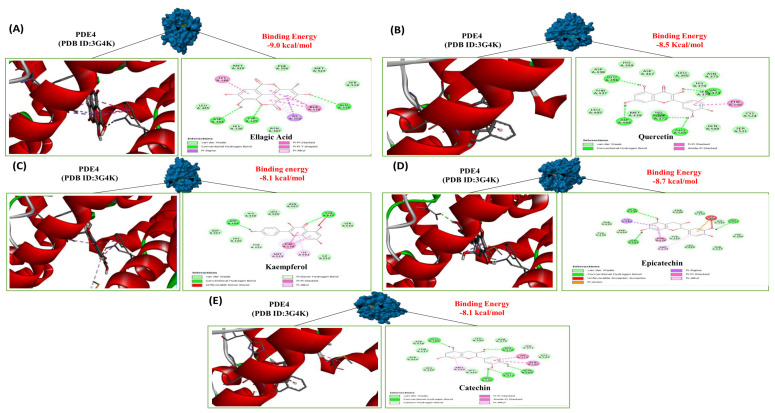
Molecular docking interaction of selected phytochemicals: (**A**) ellagic acid, (**B**) quercetin, (**C**) kaempferol, (**D**) epicatechin, and (**E**) catechin derived from ethanol extract of *P. granatum* fruit peel (EEPG) with receptor PDE4 (PDB ID:3G4K). Molecular docking analysis showed notable binding affinities of the phytochemicals towards the PDE4 receptor active site. Ellagic acid exhibited the strongest binding affinity (−9.0 kcal/mol), forming interactions, including π–sigma bonding with ILE A:502 and π–π stacked and π–π T-shaped interactions with HIS A:326 and PHE A:538. Epicatechin demonstrated considerable binding stability with a docking score of −8.7 kcal/mol. Quercetin (−8.5 kcal/mol) established conventional hydrogen bond interactions with residues, such as GLU A:396, GLY A:372, GLU A:505, VAL A:373, and ASP A:484. These findings suggest that the phytochemicals may inhibit PDE4, leading to increased cAMP signaling, enhanced insulin secretion, and improved glucose metabolism.

**Figure 8:**
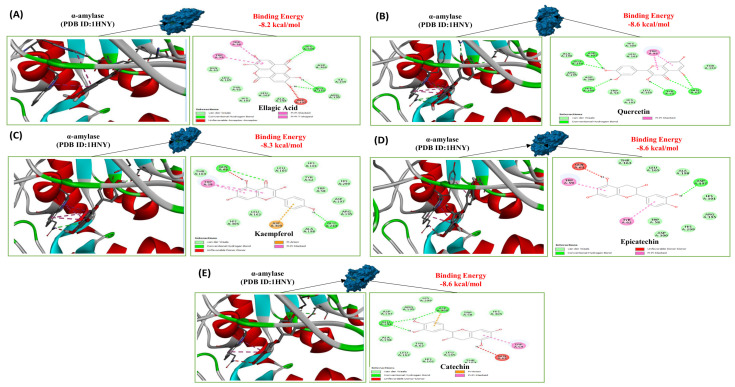
Molecular docking interaction of selected phytochemicals: (**A**) ellagic acid, (**B**) quercetin, (**C**) kaempferol, (**D**) epicatechin, and (**E**) catechin derived from ethanol extract of *P. granatum* fruit peel (EEPG) with receptor α-amylase (PDB ID:1HNY). Docking analysis demonstrated significant binding affinities of five *Punica granatum*-derived phytochemicals toward the α-amylase enzyme. Epicatechin, quercetin, and catechin exhibited binding energies of −8.6 kcal/mol and showed strong stability within the catalytic pocket by forming stable hydrogen bonds with ASP A:197, TYR A: 62, HIS A:299, GLU A: 233, ASP A:197 and ASP A:300. Kaempferol and ellagic acid displayed competitive binding affinities of −8.3 and −8.2 kcal/mol, respectively. These findings suggest that EEPG phytochemicals may inhibit α-amylase activity, thereby supporting glucose homeostasis and glycemic control.

The phytoconstituents, such as ellagic acid, quercetin, kaempferol, catechin, and epicatechin, were selected for molecular docking based on previous reports of EEPG [37]. The docking analysis showed that the interactions between ligands and receptors involved conventional hydrogen bonds, π–π stacking, π–alkyl interactions, and van der Waals forces within the binding pocket. Overall, the results showed the favorable binding of EEPG-derived phytochemicals across different protein targets. Docking interaction analysis revealed that compounds derived from EEPG showed favorable binding affinities toward SUR1 (PDB ID: 7WIT), a K_ATP_ channel regulator; PDE4 (PDB ID: 3G4K), a cAMP hydrolyzing phosphodiesterase; PI3K pathway (PDB ID: 4FLH), a modulator of cAMP levels in pancreatic β-cells; and α-amylase (PDB ID: 1HNY), a carbohydrate-digesting enzyme (Table 3; Figure 5, Figure 6, Figure 7 and Figure 8).

For the SUR1 receptor protein, ellagic acid, quercetin, kaempferol and epicatechin showed significant binding affinity energy ranges from −9.2 to −7.8 kcal/mol (Table 3; Figure 5). Similarly, for the PI3K receptor, ellagic acid again demonstrated the strongest binding affinity (−8.8 kcal/mol), while catechin, quercetin, epicatechin, and kaempferol exhibited comparatively lower but significant binding energies (−8.6 to −8.1 kcal/mol) (Table 3; Figure 6). For the PDE4 receptor protein, the binding energies range from −9.0 to −8.1 kcal/mol, representing the strongest interaction. Ellagic acid showed the highest binding affinity (−9.0 kcal/mol), followed by epicatechin and quercetin, while kaempferol and catechin exhibited comparatively lower values (Table 3; Figure 7).

In the case of α-amylase, the binding energies range from −8.6 to −8.2 kcal/mol. Unlike previous targets, multiple compounds (epicatechin, catechin, quercetin) showed identical binding energies (−8.6 kcal/mol), whereas those for kaempferol and ellagic acid were −8.3 and −8.2 kcal/mol (Table 3; Figure 8). Overall, all compounds demonstrated comparable binding affinities within a narrow range.

## 4. Discussion

Pomegranate, originating from the Lythraceae family, is widely recognized for numerous therapeutic and medicinal benefits [38]. Previous studies indicate that pomegranate peel exhibits significant anti-diabetic properties. Studies have shown that it lowers lipase and amylase levels in high-fructose diet-fed rats, effectively mitigating oxidative stress, insulin resistance, and glucose intolerance [39]. This research evaluated the anti-diabetic and β-cell proliferative activities of EEPG through in vitro and in vivo studies, investigating its mechanisms for reducing hyperglycemia and increasing insulin production from clonal pancreatic β-cells.

Ethanol extract of pomegranate fruit peel (EEPG) significantly increased insulin secretion from pancreatic β-cells in a concentration-dependent manner. To further elucidate its mechanism of action, EEPG’s effects were then investigated in the presence of high glucose, tolbutamide, membrane-depolarizing KCl concentration (30 mM), and IBMX. EEPG enhanced insulin release under these conditions. Tolbutamide, a sulphonylurea drug, is known to block K_ATP_ channels, thereby depolarizing the plasma membrane, activating L-type voltage-dependent calcium channels, and increasing intracellular Ca^2+^ influx, which triggers insulin exocytosis [40]. These findings suggest that EEPG may act, at least in part, via the K_ATP_ channel-independent pathway. EEPG-induced insulin secretion was also partially reduced by verapamil (L-type voltage-dependent Ca^2+^ channel blocker) and diazoxide (K_ATP_ channel opener), indicating a dependence on K_ATP_ channel closure and Ca^2+^ channel opening for its full insulinogenic effect [41]. Moreover, the addition of IBMX, a cAMP phosphodiesterase inhibitor, further augmented the insulin secretion induced by EEPG, suggesting that EEPG also upregulates intracellular cAMP levels [42]. Taken together, these findings suggest that EEPG may exert its insulinotropic effects through multiple mechanisms. The observed effects may also involve direct effects on exocytosis or modulation of other pathways, such as the phosphatidylinositol 3-kinase (PI3K) or adenylate cyclase pathways [43].

β-cell proliferation is a crucial mechanism for maintaining pancreatic β-cell mass and adequate insulin secretion, both of which are essential for glucose homeostasis and the prevention of diabetes [44,45]. EEPG significantly promoted β-cell proliferation at both low and high concentrations. This proliferative effect is comparable to that observed with the positive control, GLP-1, which is known to activate the cAMP/PKA signaling pathway [46]. Increased β-cell proliferation contributes to an increased β-cell mass, thereby improving insulin secretory capacity. These findings suggest that EEPG may facilitate the proliferation of insulin-producing β-cells by activating key signaling pathways, such as cAMP/PKA, PI3K/Akt, Wnt/β-catenin, or JAK-STAT [47,48].

Recent studies demonstrate that oxidative stress is an essential factor in diabetes development, by which elevated lipid peroxidation and glucose oxidation produce free radicals that damage cellular components, impair enzyme function and insulin resistance [49]. In our study, EEPG demonstrated a significant dose-dependent suppression of the marker DPPH. This effect is likely due to the presence of flavonoids in EEPG, including ellagic acid, quercetin, epicatechin and catechin, which have been shown to mitigate oxidative stress by neutralizing free radicals [50,51].

The enzymatic breakdown of starch by α-amylase and α-glucosidase, along with glucose absorption and diffusion in the gastrointestinal tract, are key pathways contributing to the pathophysiology of diabetes [52]. Our results showed that EEPG induced concentration-dependent suppression of glucose liberation from starch. These findings are consistent with prior studies on the ability of flavonoids to reduce α-amylase activity and prolong starch digestion [53].

The physiological relevance of in vitro starch digestion assays depends on the extent to which the experimental conditions mimic the major digestive processes involved in starch breakdown within the human gastrointestinal tract, including enzymatic hydrolysis, pH changes, and the release of nutrients from the food matrix [54]. The small intestine is the primary site of starch hydrolysis, making pancreatic α-amylase and brush-border glucosidases essential components of physiologically relevant digestion models [55]. Increasing evidence also suggests that salivary α-amylase plays a significant role in the initial breakdown of starch during oro-gastric digestion [56]. Therefore, the observed inhibitory effect of EEPG on starch digestion may have physiological relevance in reducing postprandial glucose release.

Furthermore, EEPG decreased both glucose diffusion and absorption. These findings further support the antidiabetic potential of EEPG by interfering with nutrient absorption and enzymatic activity [57]. However, one limitation of the present study is that the dialysis membrane model does not mimic the intestinal epithelial cell behavior. Consequently, it provides an estimate of glucose bioaccessibility or passive diffusion rather than true intestinal absorption and bioavailability [58]. Therefore, the findings should be interpreted with caution and require confirmation using more physiologically relevant models. Based on these findings, we hypothesize that the EEPG-derived compounds may play important roles and warrant further investigation in animal studies.

Obesity is a major risk factor for T2DM, characterized by high levels of non-esterified fatty acids (NEFAs) generated from adipose tissue, contributing to insulin resistance and impeding β-cell function [50]. In the present study, EEPG improved acute oral glucose tolerance and plasma insulin levels in HFDi-OM. These results are consistent with prior plant-based anti-diabetic studies, including *P. granatum* and other phytochemical-rich medicinal plants, which can improve glucose tolerance, plasma insulin response, and sustain β-cell activity [39,59,60].

Twice-daily oral administration of EEPG for 21 days substantially lowered fasting blood glucose levels in a time-dependent manner in HFDi-OM, with the higher dose exhibiting earlier and more prominent antihyperglycemic effects. These results imply that EEPG exerts a dose-dependent antihyperglycemic action, as does glibenclamide, a sulfonylurea class insulin secretagogue, which acts by improving glycemic control primarily by stimulating insulin release from pancreatic β-cells and enhancing their responsiveness to glucose [61]. HFDi-OM treated with EEPG also exhibited a consistent and long-lasting reduction in body weight, demonstrating its potential to address metabolic issues associated with obesity. The impact of EEPG on body weight may stem from improved energy utilization and decreased fat retention [62]. This is crucial, given that obesity, diabetes and non-alcoholic fatty liver disease (NAFLD) are often caused by excessive calorie consumption combined with minimal energy use, which profoundly impacts lipid metabolism [62,63]. Furthermore, EEPG also significantly reduced food and fluid consumption. These findings align with previous research on *P. granatum* peel [39].

Hyperlipidemia is a prevalent metabolic disorder characterized by elevated plasma concentrations of lipids, including cholesterol and triglycerides, arising from genetic predispositions, dietary imbalances, and a sedentary lifestyle. It is a major risk factor for atherosclerosis, cardiovascular disease, pancreatitis, and metabolic syndrome [64]. In line with previous findings on plant-based phytoconstituents with lipid-lowering effects [65], both EEPG doses significantly increased HDL-cholesterol levels while simultaneously lowering total cholesterol, LDL-cholesterol, and triglycerides. These effects suggest improved lipoprotein breakdown and enhanced reverse cholesterol transport. Furthermore, the observed rise in HDL levels might also be linked to altered hormone regulation, potentially involving thyroid-related pathways that facilitate LDL elimination from the liver [66,67].

Gastrointestinal (GI) motility refers to the coordinated contractile activity of the GI tract that governs the transit and absorption of luminal contents. Dysregulation of this process has significant metabolic implications, particularly in diabetes mellitus, where delayed gastric emptying (gastroparesis) or accelerated intestinal transit can alter postprandial glycemic responses by modulating nutrient absorption dynamics [68,69]. In the present study, administration of a higher dose of EEPG for 21 days significantly promoted intestinal transit, suggesting a reduction in glucose absorption time and contributing to its antihyperglycemic effect. These findings are consistent with prior studies on *P. granatum* peel, which have reported enhanced GI motility and smooth muscle activity attributed to its rich content of polyphenols, known to influence enteric neurotransmission and myogenic tone, thereby supporting its potential role in metabolic regulation [70].

The presence of various phytoconstituents, such as flavonoids, phenols, terpenoids, steroids and saponins, has been anticipated by preliminary phytochemical screening of EEPG. Flavonoids like epicatechin, catechin and *p*-coumaric acid improve glucose homeostasis and insulin resistance and also demonstrate antidiabetic effects by modulating glucose absorption, improving insulin signaling and secretion, and lowering fat accumulation [71]. However, further studies are warranted to identify and characterize the specific bioactive phytomolecules within EEPG, as well as to investigate the detailed molecular pathways involved in insulin secretion, β-cell proliferation, and glucose homeostasis. Additionally, in silico molecular docking analysis highlighted the binding affinity of EEPG-derived phytochemicals (ellagic acid, quercetin, kaempferol, epicatechin and catechin) towards multiple receptors. These compounds showed strong target-specific interactions, providing a molecular basis for their antidiabetic effects and guiding future experimental validation.

The sulfonylurea receptor 1 (SUR1) is a regulatory subunit of the ATP-sensitive potassium (K_ATP_) channel and plays a critical role in pancreatic β-cell insulin secretion [72]. Antidiabetic drugs such as sulfonylureas bind to SUR1, leading to closure of the K_ATP_ channel, opening of voltage-gated Ca^2+^ channels, and promotion of insulin secretion [73]. The present study depicted the strong interaction between selected phytochemicals like ellagic acid (−9.2 kcal/mol), quercetin (−8.3 kcal/mol), kaempferol (−8.1 kcal/mol), epicatechin (−7.8 kcal/mol) and the SUR1 receptor. These binding affinity values suggest that these phytochemicals may interact with SUR1, similar to sulfonylurea drugs, potentially leading to the closure of the K_ATP_ channel, membrane depolarization, and enhanced insulin secretion.

Phosphodiesterase-4 (PDE4) is present in pancreatic β-cells, where it influences insulin secretion through augmentation of cAMP signaling. It is also widely expressed in metabolically active peripheral tissues, including skeletal muscle, adipose tissue, liver, and heart, where it influences insulin sensitivity and glucose metabolism [74]. Docking analysis revealed a strong interaction between selected key phytochemicals like ellagic acid (−9.0 kcal/mol), quercetin (−8.5 kcal/mol), kaempferol (−8.1 kcal/mol), epicatechin (−8.7 kcal/mol), catechin (−8.1 kcal/mol) and the PDE4. These results indicate that these phytochemicals may act as PDE4 inhibitors, potentially elevating intracellular cAMP levels to enhance both insulin secretion and insulin sensitivity in peripheral tissues.

The PI3K signaling pathway operates as a major regulatory route leading to pancreatic beta cell proliferation through multiple interrelated downstream effectors [75]. The docking analysis revealed a strong interaction of EEPG-derived phytochemicals like ellagic acid (−8.8 kcal/mol), quercetin (−8.3 kcal/mol), kaempferol (−8.1 kcal/mol), epicatechin (−8.2 kcal/mol) and catechin (−8.6 kcal/mol) with the PI3K. Activation of PI3K by these phytochemicals may address the progressive loss of beta cell mass characteristic of type 2 diabetes.

α-Amylase catalyzes the breakdown of starch into glucose. Inhibiting its catalytic activity can reduce postprandial glucose production, which may have therapeutic benefits for individuals with diabetes [76]. The molecular docking analysis revealed a strong interaction of defined phytochemicals like ellagic acid (−8.2 kcal/mol), quercetin (−8.6 kcal/mol), kaempferol (−8.3 kcal/mol), epicatechin (−8.6 kcal/mol) and catechin (−8.6 kcal/mol) with the α-amylase. These results suggest that these phytoconstituents may act as potent α-amylase inhibitors, potentially delaying starch hydrolysis and reducing postprandial glucose spikes.

It is also shown that EEPG-derived phytochemicals like ellagic acid, quercetin, kaempferol, catechin, and epicatechin may have potential interactions with GPR40, a Kelch-like ECH-associated protein 1 (Keap1), and PPAR-α in the range of −7.1 to −8.4 kcal/mol (Table S1; Figures S1–S3). These findings suggest that these phytochemicals may modulate GPR40, Keap1 and PPAR-α, but further studies are clearly warranted.

Collectively, these molecular docking results suggest that EEPG-derived compounds may have a synergistic therapeutic mechanism that addresses diabetes pathophysiology by modulating SUR1, PI3K, PDE4 and α-amylase; however, they do not provide direct evidence of regulation of SUR1, PDE4, or PI3K. Based on the current studies, we hypothesize that these coordinated actions may contribute to closing the K_ATP_ channel (via SUR1), improving insulin signaling (via PI3K), modulating cAMP pathways (via PDE4), and controlling carbohydrate digestion (via α-amylase).

Redocking of co-crystallized ligands was not performed because PyRx/Vina is a well-validated docking platform; however, this is acknowledged as a limitation of the present study [35,77]. Another limitation of the present study is that the compounds selected for molecular docking, namely ellagic acid, quercetin, kaempferol, catechin, and epicatechin, were chosen based on previous phytochemical reports describing the composition of pomegranate peel extracts. Although these compounds have been widely reported as major constituents of *Punica granatum* peel, their presence and relative abundance in the EEPG used in the present study were not directly confirmed through chromatographic analyses such as HPLC, LC-MS, or GC-MS. Therefore, the docking results should be interpreted as predictive evidence of potential molecular interactions.

Furthermore, the mechanistic pathways predicted from the in-silico analyses require further validation through future experimental investigations. Future studies incorporating comprehensive phytochemical characterization are warranted to confirm the identity and abundance of the active constituents and to strengthen the mechanistic interpretation of the findings. Nevertheless, these findings highlight the potential of EEPG-derived phytochemicals as a multifaceted natural antidiabetic strategy capable of providing comprehensive glycemic control while potentially causing fewer side effects than single-target synthetic therapies.

## 5. Conclusions

The findings of this study highlight the promising therapeutic potential of EEPG in the dietary management of T2DM. EEPG demonstrated significant insulinotropic, antihyperglycemic, and hypolipidemic effects. In silico molecular docking further supported these findings, with key phytochemicals, including ellagic acid, quercetin, kaempferol, epicatechin, and catechin, exhibiting strong binding affinities toward SUR1, PI3K, PDE4, and α-amylase. These findings provide a molecular basis for the hypothesis that EEPG exerts insulinotropic and antidiabetic effects through interactions with multiple diabetes-related targets. However, to advance the development of effective and targeted diabetes treatments, future research should focus on identifying and characterizing the specific bioactive compounds responsible for the antidiabetic effects of EEPG.

Utilizing multi-omics approaches, such as genomics, proteomics, and metabolomics, future research is essential for a comprehensive understanding of these intricate interactions. Such strategies might be useful to uncover the precise molecular mechanisms underlying EEPG’s antidiabetic effects, identify pertinent biomarkers for its safety and efficacy, and ultimately support its development as a standardized or adjunct therapeutic option for diabetes management.

## Figures and Tables

**Table 1:** Antioxidant activity of ethanol extract of *P. granatum fruit* peel (EEPG) and L-ascorbic acid using DPPH scavenging assay.

Concentration (µg/mL)	EEPG (% Inhibition)	L-Ascorbic Acid (% Inhibition)
1.6	9.1 ± 2.7 *	11.2 ± 0.4 **
8	35.9 ± 1.8 ***	33.2 ± 0.4 ***
40	71.1 ± 3.1 ***	73.9 ± 1.1 ***
200	81.3 ± 1.2 ***	85.7 ± 1.1 ***
1000	85.9 ± 0.3 ***	92.8 ± 1.1 ***
5000	91.0 ± 0.1 ***	95.7 ± 0.1 ***

Dose-dependent free radical scavenging activity of ethanol extract of *Punica granatum* peel (EEPG) compared with L-ascorbic acid (reference antioxidant). The assay was performed at concentrations between 1.6 and 5000 µg/mL following a 30 min dark incubation. Data are expressed as mean ± SEM of percentage DPPH inhibition (*n* = 3). Levels of statistical significance versus control are denoted as * *p* < 0.05, ** *p* < 0.01, and *** *p* < 0.001.

**Table 2:** Phytochemical screening of ethanol extract of *P. granatum* fruit peel.

Group Test	Observation
Alkaloids	-
Tannins	-
Flavonoids	+
Phenols	+
Terpenoids	+
Steroids	+
Saponins	+
Glycosides	-
Reducing sugars	+

‘+’ means present; ‘-’ means not detected.

**Table 3:** Binding score of phytoconstituents with multiple proteins.

Target Protein	Ligand		Binding Residues	No. of H-Bonds
	Name	Binding Energy (kcal/mol)		
SUR1	Ellagic Acid	−9.2	Tyr377, Arg306, Gln444, Asn1293, Ser1292, Pro589, Asp310, Met1290, Arg370, Leu1289, Leu366, Gln369, Gly313, Leu373, Ile585	2
	Quercetin	−8.3	Gln444, Thr588, Asn1293, Gln369, Arg370, Glu1253, Gly313, Leu373, Gln374, Tyr377, Glu1249, Ser1292, Trp1297, Asn1296, Pro589, Gly313, Ile585,	3
	Kaempferol	−8.1	Arg1246, Arg1300, Asn1245, Ser1238, Glu1249, Leu434, Trp430, Phe433, Arg388, Met429, Ile385, Thr1242, Leu1241, Ile381	1
	Epicatechin	−7.8	Leu1027, Asp1031, Trp1297, Thr588, Thr548, Leu1149, Tyr1287, Ile1030, Leu580, Val555, Val587, Phe591, Asn547, Pro551, Ile552, His584, Arg1145	2
PI3K	Ellagic Acid	−8.8	Ser806, Lys833, Asp964, Ile963, Ile831, Tyr867, Ile879, Glu880, Phe961, Val882, Ala885, Met953, Trp812, Ile881, Met804	1
	Quercetin	−8.3	Arg690, Trp201, Tyr867, Pro866, Glu880, Asp, Gly868, Tyr787, Arg849, Ile870, Cys869, Met842, Phe698, Gln846, Leu657, Phe694	1
	Kaempferol	−8.1	Asp654, Leu657, His658, Trp201, Phe698, Arg849, Arg690, Gln846, Cys869, Phe694, Tyr787, Gly868, Asp788, Glu880	2
	Epicatechin	−8.2	Gln291, Asp654, Leu657, Trp201, His658, Arg690, Tyr787, Gln846, Arg849, Phe694, Gly868, Cys869, Ile870	1
	Catechin	−8.6	Trp812, Met953, Val882, Ala885, Ile881, Glu880, Phe961, Ile831, Met804, Lys833, Tyr963, Tyr867, Ile879, Cys869, Asp841, Leu838, Asp964	3
PDE4	Ellagic Acid	−9.0	His326, Met439, Phe506, Met523, Ser534, Gln535, Phe538, Ile502, Asn487, Tyr325, His330, Asp484, Leu485	1
	Quercetin	−8.5	Asp438, Thr437, Leu485, Glu396, His326, Asp367, Asp484, Met439, His399, Arg372, Glu505, Gln509, Ser521, Cys524, Phe506, Val373, Ser374, His370, Leu395, Asn375	5
	Kaempferol	−8.1	His326, Tyr325, Asp367, Asp494, His330, Leu485, Asn487, Gln535, Ser534, Ile542, Ile502, Phe538, Met523	1
	Epicatechin	−8.7	Gln535, Phe506, His330, Asp484, His326, Asp367, His366, Thr437, Leu485, Tyr325, Met439, Phe538, Asn487, Tyr495, Thr499, Ile502, Trp498	2
	Catechin	−8.1	Asp484, Thr437, Asp438, Glu396, Leu485, Met439, His326, Leu395, Glu505, Asn375, Gly372, His370, Gln509, Ser374, Val373, Phe506, Cys524	5
α-amylase	Ellagic Acid	−8.2	Trp59, Trp58, Gln63, Leu165, Tyr62, His101, Leu162, Ala198, Asp197, Arg195, Glu233, Ile235, Asp300, His305	2
	Quercetin	−8.6	Trp58, His290, Arg195, Asp300, Ala198, Glu233, Asp197, Leu162, His305, Trp59, Thr163, Gln63, Tyr62, Leu165, His101	4
	Kaempferol	−8.3	Thr163, Gln63, Trp59, Leu165, His305, Leu162, Asp300, Ala198, Glu233, Arg195, Asp197, His299, Trp58, Tyr62, His101	1
	Epicatechin	−8.6	Trp59, Gln63, Thr163, Leu165, Ala198, Asp197, His101, Arg195, His299, Asp300, Trp58, Tyr62	1
	Catechin	−8.6	Asp197, Arg195, His299, Asp300, Trp58, His305, Glu233, Ala198, Tyr62, Leu162, His101, Leu165, Thr163, Trp59, Gln63	3

Docking hits of EEPG-derived phytochemicals with multiple proteins. Compounds and their binding affinity in kcal/mol are mentioned. The more negative values indicate stronger binding affinity.

## Data Availability

Data are not publicly accessible due to institutional and ethical restrictions. However, it can be obtainable to qualified researchers upon reasonable request to the corresponding author. Any data sharing will be considered in compliance with applicable regulations and institutional policies.

## Supplementary Materials

The following supporting information can be downloaded at: https://www.mdpi.com/article/10.3390/cimb48070670/s1.

## References

[1] Alam S., Sarker M.M.R., Sultana T.N., Chowdhury M.N.R., Rashid M.A., Chaity N.I., Zhao C., Xiao J., Hafez E.E., Khan S.A. (2022). Antidiabetic Phytochemicals from Medicinal Plants: Prospective Candidates for New Drug Discovery and Development. Front. Endocrinol..

[2] Ali A.M., Moqbel M.S., Al-Hizab F.A. (2022). Effect of Momordica Charantia on Insulin Immune-Reactive Pancreatic Beta Cells and Blood Glucose Levels in Streptozotocin-Induced Diabetic Rats. J. Nutr. Sci. Vitaminol..

[3] Özpak Akkuş Ö., Metin U., Çamlık Z. (2023). The Effects of Pomegranate Peel Added Bread on Anthropometric Measurements, Metabolic and Oxidative Parameters in Individuals with Type 2 Diabetes: A Double-Blind, Randomized, Placebo-Controlled Study. Nutr. Res. Pract..

[4] Genitsaridi I., Salpea P., Salim A., Sajjadi S.F., Tomic D., James S., Thirunavukkarasu S., Issaka A., Chen L., Basit A. (2026). 11th Edition of the IDF Diabetes Atlas: Global, Regional, and National Diabetes Prevalence Estimates for 2024 and Projections for 2050. Lancet Diabetes Endocrinol..

[5] Maphetu N., Unuofin J.O., Masuku N.P., Olisah C., Lebelo S.L. (2022). Medicinal Uses, Pharmacological Activities, Phytochemistry, and the Molecular Mechanisms of *Punica granatum* L. (Pomegranate) Plant Extracts: A Review. Biomed. Pharmacother..

[6] Janssen J.A.M.J.L. (2021). Hyperinsulinemia and Its Pivotal Role in Aging, Obesity, Type 2 Diabetes, Cardiovascular Disease and Cancer. Int. J. Mol. Sci..

[7] Willard F.S., Douros J.D., Gabe M.B.N., Showalter A.D., Wainscott D.B., Suter T.M., Capozzi M.E., van der Velden W.J.C., Stutsman C., Cardona G.R. (2020). Tirzepatide Is an Imbalanced and Biased Dual GIP and GLP-1 Receptor Agonist. JCI Insight.

[8] Nauck M.A., D’Alessio D.A. (2022). Tirzepatide, a Dual GIP/GLP-1 Receptor Co-Agonist for the Treatment of Type 2 Diabetes with Unmatched Effectiveness Regrading Glycaemic Control and Body Weight Reduction. Cardiovasc. Diabetol..

[9] Tirzepatide Cost in 2026: Brand vs. Compounded Pricing. https://healthymealsincentives.org/tirzepatide-cost/.

[10] Keller A.C., Ma J., Kavalier A., He K., Brillantes A.-M.B., Kennelly E.J. (2011). Saponins from the Traditional Medicinal Plant *Momordica Charantia* Stimulate Insulin Secretion In Vitro. Phytomedicine.

[11] Aslam T., Arif A., Arshad S., Muccee F., Ahmad K., Iqbal M.O., Khalil U., Razak S., Afsar T., Almajwal A. (2024). Discovering the Anti-Diabetic Potential of Pomegranate Peel Metabolites by Examining Molecular Interplay with the Thioredoxin-Interacting Protein. Front. Med..

[12] Eghbali S., Askari S.F., Avan R., Sahebkar A. (2021). Therapeutic Effects of Punica Granatum (Pomegranate): An Updated Review of Clinical Trials. J. Nutr. Metab..

[13] Arun N., Singh D.P. (2012). Punica Granatum: A Review on Pharmacological and Therapeutic Properties. J. Pharm. Sci. Res..

[14] El-Hadary A.E., Ramadan M.F. (2019). Phenolic Profiles, Antihyperglycemic, Antihyperlipidemic, and Antioxidant Properties of Pomegranate (Punica Granatum) Peel Extract. J. Food Biochem..

[15] Olvera-Sandoval C., Fabela-Illescas H.E., Fernández-Martínez E., Ortiz-Rodríguez M.A., Cariño-Cortés R., Ariza-Ortega J.A., Hernández-González J.C., Olivo D., Valadez-Vega C., Belefant-Miller H. (2022). Potential Mechanisms of the Improvement of Glucose Homeostasis in Type 2 Diabetes by Pomegranate Juice. Antioxidants.

[16] Mokgalaboni K., Dlamini S., Phoswa W.N., Modjadji P., Lebelo S.L. (2023). The Impact of Punica Granatum Linn and Its Derivatives on Oxidative Stress, Inflammation, and Endothelial Function in Diabetes Mellitus: Evidence from Preclinical and Clinical Studies. Antioxidants.

[17] Mo Y., Ma J., Gao W., Zhang L., Li J., Li J., Zang J. (2022). Pomegranate Peel as a Source of Bioactive Compounds: A Mini Review on Their Physiological Functions. Front. Nutr..

[18] Nunes A.R., Alves G., Falcão A., Lopes J.A., Silva L.R. (2025). Phenolic Acids from Fruit By-Products as Therapeutic Agents for Metabolic Syndrome: A Review. Int. J. Mol. Sci..

[19] Bouyahya A., Balahbib A., Khalid A., Makeen H.A., Alhazmi H.A., Albratty M., Hermansyah A., Ming L.C., Goh K.W., Omari N.E. (2024). Clinical Applications and Mechanism Insights of Natural Flavonoids against Type 2 Diabetes Mellitus. Heliyon.

[20] Russo B., Picconi F., Malandrucco I., Frontoni S. (2019). Flavonoids and Insulin-Resistance: From Molecular Evidences to Clinical Trials. Int. J. Mol. Sci..

[21] Ramya S., Narayanan V., Ponnerulan B., Saminathan E., Veeranan U. (2020). Potential of Peel Extracts of Punica Granatum and Citrus Aurantifolia on Alloxan-Induced Diabetic Rats. Beni-Suef Univ. J. Basic Appl. Sci..

[22] Ansari P., Flatt P.R., Harriott P., Abdel-Wahab Y.H.A. (2022). Insulin Secretory and Antidiabetic Actions of Heritiera Fomes Bark Together with Isolation of Active Phytomolecules. PLoS ONE.

[23] Ansari P., Hannan J.M.A., Seidel V., Abdel-Wahab Y.H.A. (2022). Polyphenol-Rich Leaf of Annona Squamosa Stimulates Insulin Release from BRIN-BD11 Cells and Isolated Mouse Islets, Reduces (CH_2_O)n Digestion and Absorption, and Improves Glucose Tolerance and GLP-1 (7-36) Levels in High-Fat-Fed Rats. Metabolites.

[24] Ansari P., Islam S.S., Ali A., Masud M.S.R., Reberio A.D., Khan J.T., Hannan J.M.A., Flatt P.R., Abdel-Wahab Y.H.A. (2025). Insulinotropic and Beta-Cell Proliferative Effects of Unripe Artocarpus Heterophyllus Extract Ameliorate Glucose Dysregulation in High-Fat-Fed Diet-Induced Obese Mice. Diabetology.

[25] Ansari P., Khan J.T., Soultana M., Hunter L., Chowdhury S., Priyanka S.K., Paul S.R., Flatt P.R., Abdel-Wahab Y.H.A. (2024). Insulin Secretory Actions of Polyphenols of Momordica Charantia Regulate Glucose Homeostasis in Alloxan-Induced Type 2 Diabetic Rats. RPS Pharm. Pharmacol. Rep..

[26] Ansari P., Flatt P.R., Harriott P., Abdel-Wahab Y.H.A. (2020). Evaluation of the Antidiabetic and Insulin Releasing Effects of *A. squamosa*, Including Isolation and Characterization of Active Phytochemicals. Plants.

[27] López-Soldado I., Guinovart J.J., Duran J. (2021). Increasing Hepatic Glycogen Moderates the Diabetic Phenotype in Insulin-Deficient Akita Mice. J. Biol. Chem..

[28] Ansari P., Hannan J.M.A., Choudhury S.T., Islam S.S., Talukder A., Seidel V., Abdel-Wahab Y.H.A. (2022). Antidiabetic Actions of Ethanol Extract of Camellia Sinensis Leaf Ameliorates Insulin Secretion, Inhibits the DPP-IV Enzyme, Improves Glucose Tolerance, and Increases Active GLP-1 (7–36) Levels in High-Fat-Diet-Fed Rats. Medicines.

[29] Ansari P., Islam S.S., Akther S., Khan J.T., Shihab J.A., Abdel-Wahab Y.H.A. (2023). Insulin Secretory Actions of Ethanolic Extract of Acacia Arabica Bark in High Fat-Fed Diet-Induced Obese Type 2 Diabetic Rats. Biosci. Rep..

[30] Ayodele P.F., Bamigbade A., Bamigbade O.O., Adeniyi I.A., Tachin E.S., Seweje A.J., Farohunbi S.T. (2023). Illustrated Procedure to Perform Molecular Docking Using PyRx and Biovia Discovery Studio Visualizer: A Case Study of 10kt with Atropine. Prog. Drug Discov. Biomed. Sci..

[31] Wang M., Wu J.-X., Chen L. (2022). Structural Insights Into the High Selectivity of the Anti-Diabetic Drug Mitiglinide. Front. Pharmacol..

[32] Burgin A.B., Magnusson O.T., Singh J., Witte P., Staker B.L., Bjornsson J.M., Thorsteinsdottir M., Hrafnsdottir S., Hagen T., Kiselyov A.S. (2010). Design of Phosphodiesterase 4D (PDE4D) Allosteric Modulators for Enhancing Cognition with Improved Safety. Nat. Biotechnol..

[33] Norman M.H., Andrews K.L., Bo Y.Y., Booker S.K., Caenepeel S., Cee V.J., D’Angelo N.D., Freeman D.J., Herberich B.J., Hong F.-T. (2012). Selective Class I Phosphoinositide 3-Kinase Inhibitors: Optimization of a Series of Pyridyltriazines Leading to the Identification of a Clinical Candidate, AMG 511. J. Med. Chem..

[34] Brayer G.D., Luo Y., Withers S.G. (1995). The Structure of Human Pancreatic α-Amylase at 1.8 Å Resolution and Comparisons with Related Enzymes. Protein Sci..

[35] Trott O., Olson A.J. (2010). AutoDock Vina: Improving the Speed and Accuracy of Docking with a New Scoring Function, Efficient Optimization, and Multithreading. J. Comput. Chem..

[36] Liu Y., Yang X., Gan J., Chen S., Xiao Z.-X., Cao Y. (2022). CB-Dock2: Improved Protein–Ligand Blind Docking by Integrating Cavity Detection, Docking and Homologous Template Fitting. Nucleic Acids Res..

[37] Tumbarski Y., Ivanov I., Vrancheva R., Mazova N., Nikolova K. (2025). Pomegranate Peels: A Promising Source of Biologically Active Compounds with Potential Application in Cosmetic Products. Cosmetics.

[38] Royapuram Parthasarathy P., Ilammaran Varshan E., Shanmugam R. (2023). In Vitro Anti-Diabetic Activity of Pomegranate Peel Extract-Mediated Strontium Nanoparticles. Cureus.

[39] Amri Z., Ben Khedher M.R., Zaibi M.S., Kharroubi W., Turki M., Ayadi F., Hammami M. (2020). Anti-Diabetic Effects of Pomegranate Extracts in Long-Term High Fructose-Fat Fed Rats. Clin. Phytosci..

[40] Pratt E.P.S., Salyer A.E., Guerra M.L., Hockerman G.H. (2016). Ca2+ Influx through L-Type Ca2+ Channels and Ca2+-Induced Ca2+ Release Regulate cAMP Accumulation and Epac1-Dependent ERK 1/2 Activation in INS-1 Cells. Mol. Cell. Endocrinol..

[41] Chen H., Zhang D., Hua Ren J., Ping Chao S. (2013). Effects of L-Type Calcium Channel Antagonists Verapamil and Diltiazem on fKv1.4ΔN Currents in Xenopus Oocytes. Iran. J. Pharm. Res..

[42] Ansari P., Flatt P.R., Harriott P., Abdel-Wahab Y.H.A. (2021). Insulinotropic and Antidiabetic Properties of Eucalyptus Citriodora Leaves and Isolation of Bioactive Phytomolecules. J. Pharm. Pharmacol..

[43] Hannan J.M.A., Marenah L., Ali L., Rokeya B., Flatt P.R., Abdel-Wahab Y.H.A. (2006). Ocimum Sanctum Leaf Extracts Stimulate Insulin Secretion from Perfused Pancreas, Isolated Islets and Clonal Pancreatic β-Cells. J. Endocrinol..

[44] Kulkarni R.N., Mizrachi E.-B., Ocana A.G., Stewart A.F. (2012). Human β-Cell Proliferation and Intracellular Signaling. Diabetes.

[45] Dludla P.V., Mabhida S.E., Ziqubu K., Nkambule B.B., Mazibuko-Mbeje S.E., Hanser S., Basson A.K., Pheiffer C., Kengne A.P. (2023). Pancreatic β-Cell Dysfunction in Type 2 Diabetes: Implications of Inflammation and Oxidative Stress. World J. Diabetes.

[46] Ali A., Khan D., Dubey V., Tarasov A.I., Flatt P.R., Irwin N. (2024). Comparative Effects of GLP-1 and GLP-2 on Beta-Cell Function, Glucose Homeostasis and Appetite Regulation. Biomolecules.

[47] Zheng Z., Zong Y., Ma Y., Tian Y., Pang Y., Zhang C., Gao J. (2024). Glucagon-like Peptide-1 Receptor: Mechanisms and Advances in Therapy. Signal Transduct. Target. Ther..

[48] Stewart A.F., Hussain M.A., García-Ocaña A., Vasavada R.C., Bhushan A., Bernal-Mizrachi E., Kulkarni R.N. (2015). Human β-Cell Proliferation and Intracellular Signaling: Part 3. Diabetes.

[49] Maritim A.C., Sanders R.A., Watkins J.B. (2003). Diabetes, Oxidative Stress, and Antioxidants: A Review. J. Biochem. Mol. Toxicol..

[50] Ansari P., Akther S., Hannan J.M.A., Seidel V., Nujat N.J., Abdel-Wahab Y.H.A. (2022). Pharmacologically Active Phytomolecules Isolated from Traditional Antidiabetic Plants and Their Therapeutic Role for the Management of Diabetes Mellitus. Molecules.

[51] Sharma O.P., Bhat T.K. (2009). DPPH Antioxidant Assay Revisited. Food Chem..

[52] Ojah E.O., Moronkola D.O., Akintunde A.-A.M., Ojah E.O., Moronkola D.O., Akintunde A.-A.M. (2020). α-Amylase and α-Glucosidase Antidiabetic Potential of Ten Essential Oils from *Calophyllum inophyllum* Linn. Iberoam. J. Med..

[53] Proença C., Freitas M., Ribeiro D., Tomé S.M., Oliveira E.F.T., Viegas M.F., Araújo A.N., Ramos M.J., Silva A.M.S., Fernandes P.A. (2019). Evaluation of a Flavonoids Library for Inhibition of Pancreatic α-Amylase towards a Structure–Activity Relationship. J. Enzym. Inhib. Med. Chem..

[54] Li H., Liu G., Liu Y., Yuan P., Liu S., Yan M., Zou Y., Wang H., Zhang T., Duan S. (2025). Effects of Different Drying Processes on Bioactive Components, Volatile Compounds, and In Vitro Inhibition of Starch Digestion in Mulberry Leaf Extracts. Foods.

[55] Zhou H., Tan Y., McClements D.J. (2023). Applications of the INFOGEST In Vitro Digestion Model to Foods: A Review. Annu. Rev. Food Sci. Technol..

[56] Freitas D., Le Feunteun S. (2019). Oro-Gastro-Intestinal Digestion of Starch in White Bread, Wheat-Based and Gluten-Free Pasta: Unveiling the Contribution of Human Salivary α-Amylase. Food Chem..

[57] Ansari P., Flatt P.R., Harriott P., Abdel-Wahab Y.H.A. (2021). Anti-Hyperglycaemic and Insulin-Releasing Effects of Camellia Sinensis Leaves and Isolation and Characterisation of Active Compounds. Br. J. Nutr..

[58] Mackie A., Mulet-Cabero A.-I., Torcello-Gómez A. (2020). Simulating Human Digestion: Developing Our Knowledge to Create Healthier and More Sustainable Foods. Food Funct..

[59] Wickramasinghe A.S.D., Kalansuriya P., Attanayake A.P. (2021). Herbal Medicines Targeting the Improved β-Cell Functions and β-Cell Regeneration for the Management of Diabetes Mellitus. Evid. Based Complement. Altern. Med..

[60] Takahama U., Hirota S. (2018). Interactions of Flavonoids with α-Amylase and Starch Slowing down Its Digestion. Food Funct..

[61] Basabe J.C., Farina J.M.S., Chieri R.A. (1976). Studies on the Dynamics and Mechanism of Glibenclamide-Induced Insulin Secretion. Horm. Metab. Res..

[62] Choe S.S., Huh J.Y., Hwang I.J., Kim J.I., Kim J.B. (2016). Adipose Tissue Remodeling: Its Role in Energy Metabolism and Metabolic Disorders. Front. Endocrinol..

[63] Pichiah P.B.T., Moon H.-J., Park J.-E., Moon Y.-J., Cha Y.-S. (2012). Ethanolic Extract of Seabuckthorn (*Hippophae rhamnoides* L.) Prevents High-Fat Diet–Induced Obesity in Mice through down-Regulation of Adipogenic and Lipogenic Gene Expression. Nutr. Res..

[64] Hill M., Bordoni B. (2023). Hyperlipidemia. StatPearls.

[65] Zarfeshany A., Asgary S., Javanmard S.H. (2014). Potent Health Effects of Pomegranate. Adv. Biomed. Res..

[66] Mokhtari I., Shahat A.A., Noman O.M., Milenkovic D., Amrani S., Harnafi H. (2024). Effects of *Cynara scolymus* L. Bract Extract on Lipid Metabolism Disorders Through Modulation of HMG-CoA Reductase, Apo A-1, PCSK-9, p-AMPK, SREBP-2, and CYP2E1 Expression. Metabolites.

[67] Sinha R.A., Singh B.K., Yen P.M. (2018). Direct Effects of Thyroid Hormones on Hepatic Lipid Metabolism. Nat. Rev. Endocrinol..

[68] Sanders K.M., Koh S.D., Ro S., Ward S.M. (2012). Regulation of Gastrointestinal Motility—Insights from Smooth Muscle Biology. Nat. Rev. Gastroenterol. Hepatol..

[69] Pal P., Pramanik S., Ray S. (2021). Editor’s Pick: Disorders of Gastrointestinal Motility in Diabetes Mellitus: An Unattended Borderline Between Diabetologists and Gastroenterologists. Diabetes.

[70] Guo Y., Wang L., Huang J.-Q., Lu M.-W., Yang S.-H. (2025). Valorization of Pomegranate Peel: Mechanisms and Clinical Applications in Irritable Bowel Syndrome Management. Int. J. Mol. Sci..

[71] Singh J., Kaur H.P., Verma A., Chahal A.S., Jajoria K., Rasane P., Kaur S., Kaur J., Gunjal M., Ercisli S. (2023). Pomegranate Peel Phytochemistry, Pharmacological Properties, Methods of Extraction, and Its Application: A Comprehensive Review. ACS Omega.

[72] Martin G.M., Patton B.L., Shyng S.-L. (2023). KATP Channels in Focus: Progress toward a Structural Understanding of Ligand Regulation. Curr. Opin. Struct. Biol..

[73] Tucker S.J., Ashcroft F.M. (1998). A Touching Case of Channel Regulation: The ATP-Sensitive K+ Channel. Curr. Opin. Neurobiol..

[74] Tengholm A. (2012). Cyclic AMP Dynamics in the Pancreatic β-Cell. Upsala J. Med. Sci..

[75] Wittenberg A.D., Azar S., Klochendler A., Stolovich-Rain M., Avraham S., Birnbaum L., Gallimidi A.B., Katz M., Dor Y., Meyuhas O. (2016). Phosphorylated Ribosomal Protein S6 Is Required for Akt-Driven Hyperplasia and Malignant Transformation, but Not for Hypertrophy, Aneuploidy and Hyperfunction of Pancreatic β-Cells. PLoS ONE.

[76] Khadayat K., Marasini B.P., Gautam H., Ghaju S., Parajuli N. (2020). Evaluation of the Alpha-Amylase Inhibitory Activity of Nepalese Medicinal Plants Used in the Treatment of Diabetes Mellitus. Clin. Phytosci..

[77] Dallakyan S., Olson A.J., Hempel J.E., Williams C.H., Hong C.C. (2015). Small-Molecule Library Screening by Docking with PyRx. Chemical Biology: Methods and Protocols.

