# Supplementary material for: Peel of Pomegranate Fruit (Punica granatum) Improves Glucose Homeostasis in Obese Mice: An Integrated In Vitro, In Vivo, and In Silico Molecular Docking Study
Source: Curr Issues Mol Biol. 2026 Jun 29;48(7):670. doi: 10.3390/cimb48070670 (PMC13407034; doi:10.3390/cimb48070670)
Supplement: Supplementary file 1 [file cimb-48-00670-s001.zip › cimb-4376970-supplementary.pdf]

Supplementary (S): Peel of pomegranate fruit (*Punica granatum*) Improves Glucose Homeostasis in Obese Mice: An Integrated *In Vitro*, *In Vivo*, and In Silico Molecular Docking Study

Table S1: Binding score of phytoconstituents with multiple proteins

Target Protein	Ligand		Binding Residues	No. of H-bonds
	Name	Binding Energy (Kcal/mol)		
GPR40	Ellagic Acid	-8.0	Leu2253, Pro2251, Ser2247, Pro179, Gly2254, Gly2255, Gly180, Ala179, Arg183, Arg2258, Glu172	4
	Quercetin	-7.1	Ile197, Ile130, Gly95, Ala98, Ala102, Val126, Tyr44, Ala99, Leu106, Ala103, Ser123, Tyr114, Phe117, Tyr122, Arg118, Pro40	2
	Kaempferol	-7.1	Pro80, Val81, Ala83, Val84, Phe87, Leu135, Leu138, Gly139, Phe142, Leu158	1
	Epicatechin	-7.2	Pro176, Gly180, Arg183, Glu172, Ala179, Ser2247, Asn2244, Ser2243, Phe2248, Leu2262, Arg2258, Gly2255, Lys2259	0
Keap1	Ellagic Acid	-8.1	Met34, Gly143, Leu147, Glu29, Ala28, Phe207, Gly139, Asp140, Arg136, Phe214, Asn210, Thr135, Tyr114	2
	Quercetin	-8.0	Gln38, Met34, Arg35, Glu29, Ala28, His25, Phe207, Ser142, Gly139, Arg136, Asn210, Asp140, Thr135	2
	Epicatechin	-7.8	Gln38, Val146, Glu29, Leu147, Phe214, Met34, Ala28, Gly139, Phe207, Arg136, Asn210, Asp140, Thr135	2
	Catechin	-7.6	Arg136, Asp140, Thr135, Gly139, Leu147, Met34, Val146, Gln38, His25, Glu29, Phe214, Ala28, Asn210, Phe207	4
PPAR- α	Ellagic Acid	-8.4	Asn221, Ser323, Met320, Met220, Val324, Leu331, Val332, Ala333, Tyr334, Thr279, Glu282, Thr283, Phe218, Glu286	2
	Quercetin	-7.5	Tyr334, Gly335, Pro238, Asn336, Pro237, Asn236, Ile228, Ser234, Lys232, Gly231, Ser230, Val227, Ala233	6
	Kaempferol	-7.2	Ala250, Leu247, Ile339, Ile241, Glu251, Val332, Leu254, Cys275, Val255, Ala333, Thr279, Tyr334	0
	Epicatechin	-7.4	Met355, Met330, Ile339, Leu344, Val332, Ala250, Val255, Ala333, Leu254, Glu251, Leu247, Ile241, Ile272, Thr279, Cys276, Cys275	0
	Catechin	-7.7	Glu251, Cys275, Leu254, Ile241, Leu247, Ala250, Val255, Ala333, Thr279, Cys276, Ile272, Ile339, Met355, Met330, Val332, Leu344	2

Docking hits of EEPG-derived phytochemicals with multiple proteins. Compounds and their binding affinities in kcal/mol are mentioned, where more negative values indicate stronger binding.

Figure Legends

Figure S1: Molecular docking interaction of selected phytochemicals: (A) Ellagic Acid, (B) Quercetin, (C) Kaempferol, and (D) Epicatechin derived from ethanol extract of *P. granatum* fruit peel (EEPG) with receptor GPR40 (PDB ID:5TZY).

Molecular docking analysis showed notable binding affinities of the phytochemicals towards the GPR40 receptor. Ellagic acid (-8.0 kcal/mol) exhibited a stable interaction network, forming hydrogen bonds with GLU A:172 and PRO A:2251. Epicatechin (-7.2 kcal/mol) showed multiple strong non-covalent interactions, including π-cation and π-alkyl bonds with ARG A:183, ARG A:2258, and ALA A:179. Quercetin displayed conventional hydrogen bonding with ALA A:99 and TYR A:114, along with π-alkyl interactions involving ALA A:102, ALA A:99, and VAL A:126. Kaempferol exhibited π-alkyl interactions with PRO A:80, LEU A:138, and VAL A:84, in addition to a conventional hydrogen bond with LEU A:135. These interactions suggest the potential modulatory effects of *Punica granatum* phytochemicals on GPR40, supporting their possible role in enhancing insulin secretion and maintaining glucose homeostasis.

Figure S2: Molecular docking interaction of selected phytochemicals: (A) Ellagic Acid, (B) Quercetin, (C) Kaempferol, (D) Epicatechin, and (E) Catechin derived from ethanol extract of *P. granatum* fruit peel (EEPG) with Keap1 protein (PDB ID:1N3U).

Docking analysis revealed strong binding affinities of Punica granatum-derived phytochemicals toward the Keap1 protein active site. Ellagic acid exhibited the strongest binding affinity (-8.1 kcal/mol), forming conventional hydrogen bonds with ARG A:136 and ALA A:28, along with π-alkyl interactions with MET A:34 and ALA A:28. Quercetin (-8.0 kcal/mol) exhibited π-alkyl interactions with MET A:34 and hydrogen bonding interactions with ASP A:140 and GLU A:29. Epicatechin (-7.8 kcal/mol) has hydrogen bonds with ARG A:136 and ASP A:140. Additionally, Catechin showed π-alkyl interactions involving MET A:34 and LEU A:147, hydrogen bonds with GLN A:38, PHE A:207, THR A:135, and ARG A:136. These binding interactions demonstrate that phytochemicals derived from EEPG may bind to the Keap1 protein efficiently, potentially activating Nrf2 and reducing oxidative stress.

Figure S3: Molecular docking interaction of selected phytochemicals: (A) Ellagic Acid, (B) Quercetin, (C) Kaempferol, (D) Epicatechin, and (E) Catechin derived from ethanol extract of *P. granatum* fruit peel (EEPG) with receptor PPARα (PDB ID:2P54).

Docking analysis demonstrated favorable binding affinities of the phytochemicals toward the PPARα protein. Ellagic acid (-8.4 kcal/mol) exhibited the highest binding affinity, forming hydrogen bonds with MET A:220 and GLU A:286. Catechin (-7.7 kcal/mol) demonstrated stable binding through conventional hydrogen bonds with LEU A:247 and MET A:330, along with π-alkyl and π-sigma interactions involving ILE A:339, ALA A:333, VAL A:255, ALA A:250, and VAL A:332. Quercetin showed multiple hydrogen bonding interactions with TYR A:334, GLY A:335, ASN A:336, ILE A:228, and SER A:239. Epicatechin exhibited hydrophobic interaction profiles characterized by π-sigma and π-alkyl contacts with VAL A:332, MET A:330, ALA A:250, ILE A:339, ALA A:333, and VAL A:255. These interaction patterns suggest that the phytochemicals bind favorably within the PPARα active site, indicating their potential role in modulating lipid metabolism and energy homeostasis.

Figure S1:

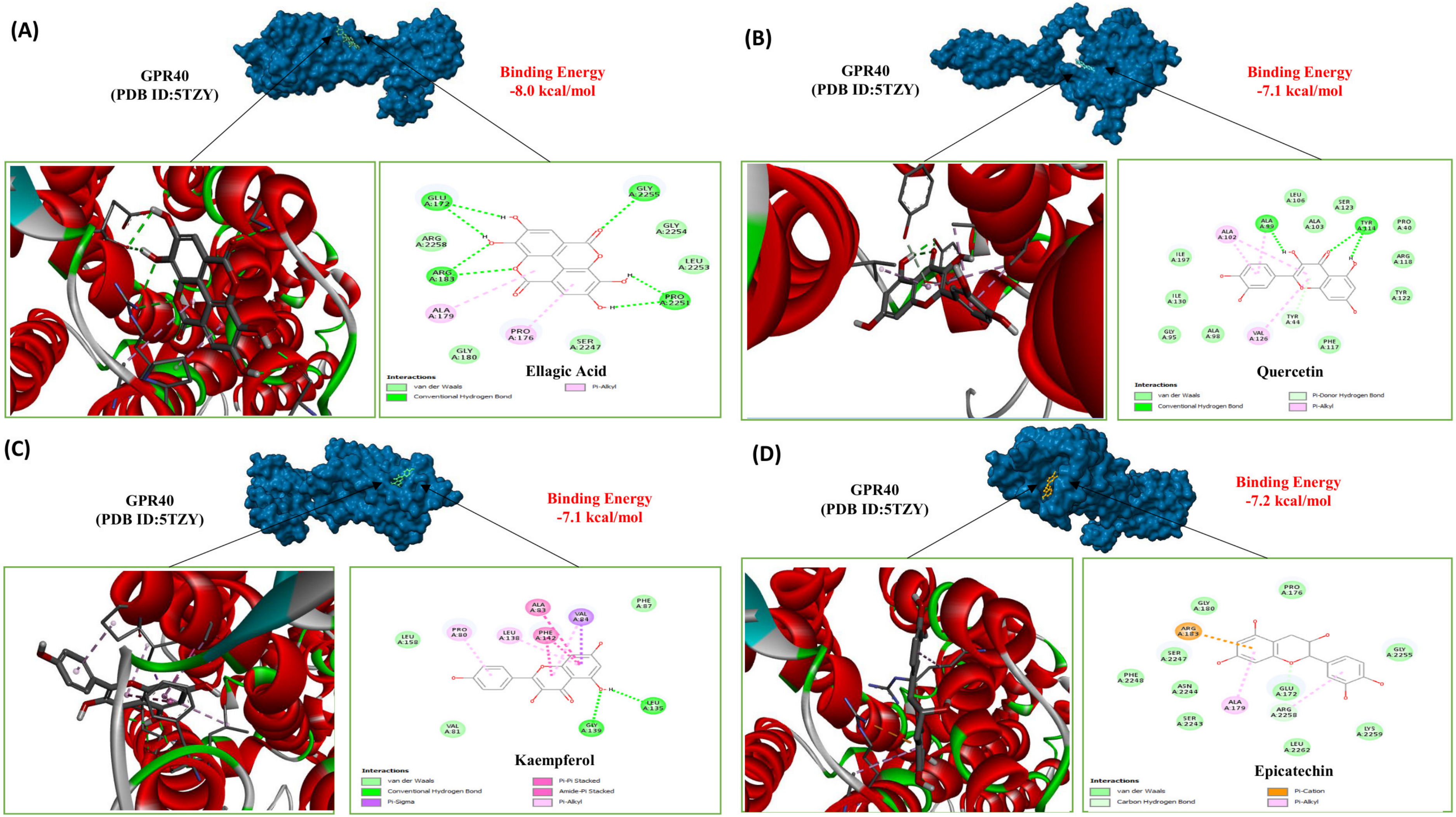
(A)
GPR40
(PDB ID:5TZY)
Binding Energy
-8.0 kcal/mol
Ellagic Acid
Interactions
van der Waals
Conventional Hydrogen Bond
Pi-Alkyl
(B)
GPR40
(PDB ID:5TZY)
Binding Energy
-7.1 kcal/mol
Quercetin
Interactions
van der Waals
Conventional Hydrogen Bond
Pi-Donor Hydrogen Bond
Pi-Alkyl
(C)
GPR40
(PDB ID:5TZY)
Binding Energy
-7.1 kcal/mol
Kaempferol
Interactions
van der Waals
Conventional Hydrogen Bond
Pi-Sigma
Pi-Pi Stacked
Amide-Pi Stacked
Pi-Alkyl
(D)
GPR40
(PDB ID:5TZY)
Binding Energy
-7.2 kcal/mol
Epicatechin
Interactions
van der Waals
Carbon Hydrogen Bond
Pi-Cation
Pi-Alkyl

Figure S2:

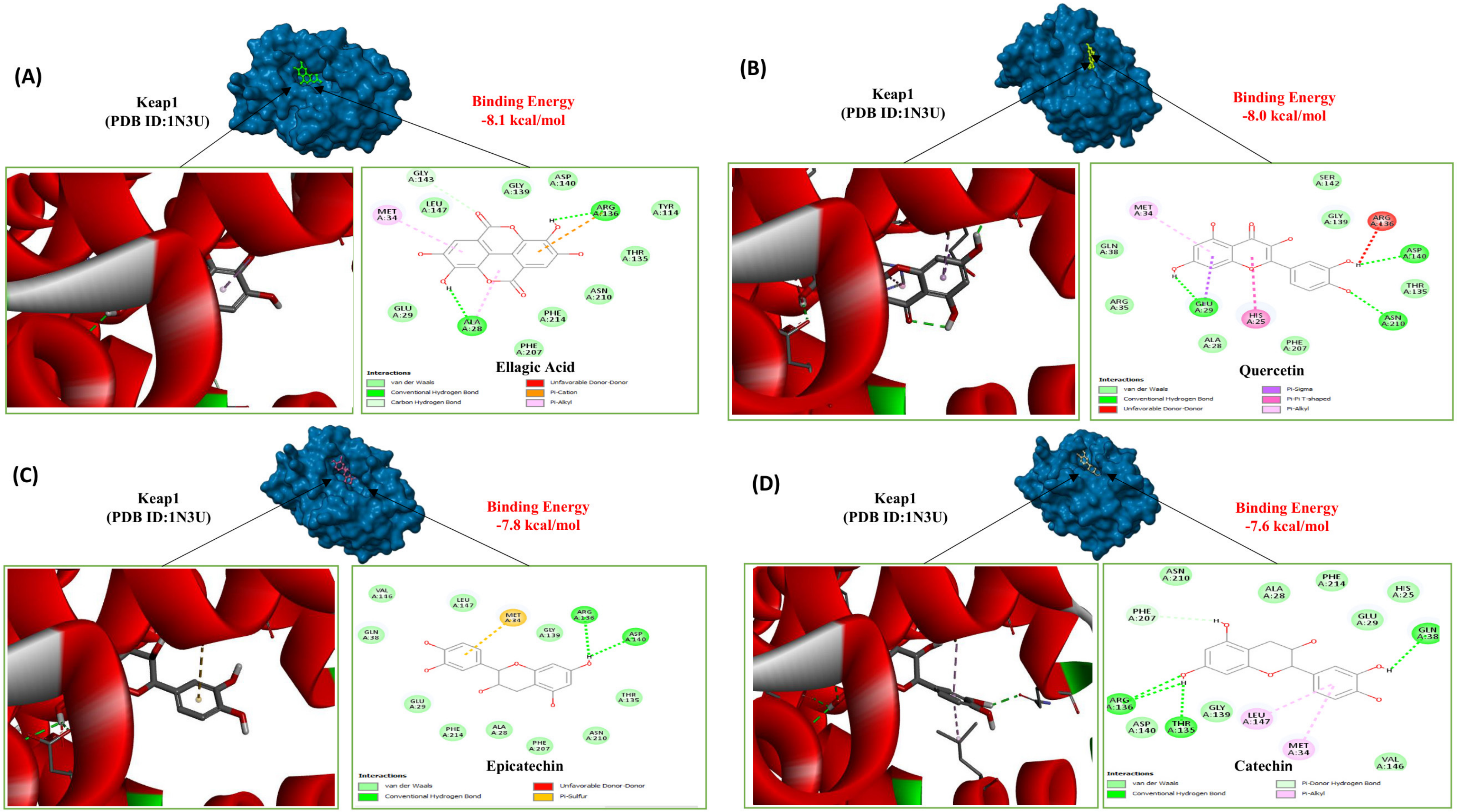
(A)
Keap1
(PDB ID:1N3U)
Binding Energy
-8.1 kcal/mol
Ellagic Acid
Interactions
van der Waals
Conventional Hydrogen Bond
Carbon Hydrogen Bond
Unfavorable Donor-Donor
Pi-Cation
Pi-Alkyl
(B)
Keap1
(PDB ID:1N3U)
Binding Energy
-8.0 kcal/mol
Quercetin
Interactions
van der Waals
Conventional Hydrogen Bond
Unfavorable Donor-Donor
Pi-Sigma
Pi-Pi T-shaped
Pi-Alkyl
(C)
Keap1
(PDB ID:1N3U)
Binding Energy
-7.8 kcal/mol
Epicatechin
Interactions
van der Waals
Conventional Hydrogen Bond
Unfavorable Donor-Donor
Pi-Sulfur
(D)
Keap1
(PDB ID:1N3U)
Binding Energy
-7.6 kcal/mol
Catechin
Interactions
van der Waals
Conventional Hydrogen Bond
Pi-Donor Hydrogen Bond
Pi-Alkyl

Figure S3:

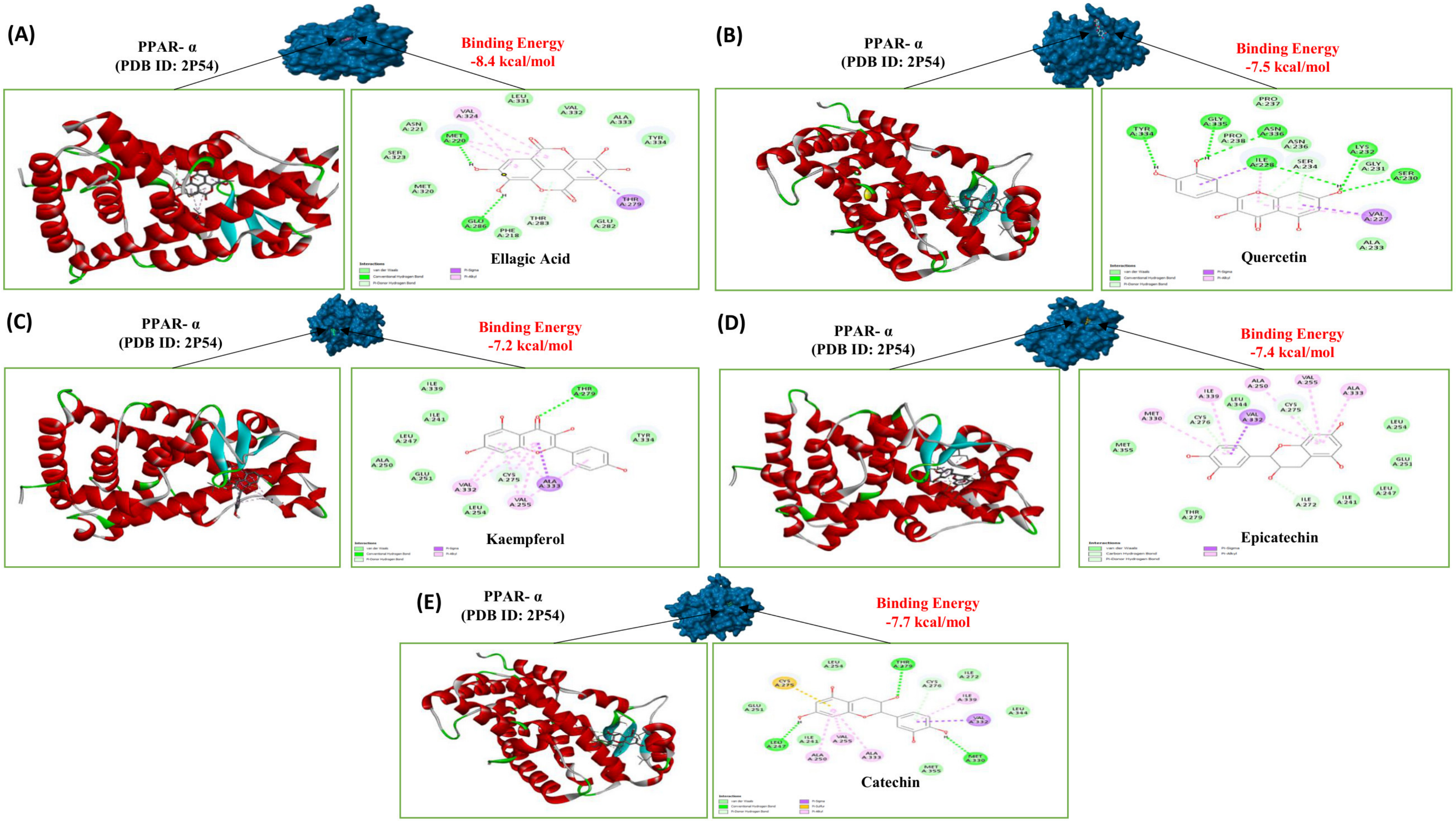
(A)
PPAR- α
(PDB ID: 2P54)
Binding Energy
-8.4 kcal/mol
Ellagic Acid
(B)
PPAR- α
(PDB ID: 2P54)
Binding Energy
-7.5 kcal/mol
Quercetin
(C)
PPAR- α
(PDB ID: 2P54)
Binding Energy
-7.2 kcal/mol
Kaempferol
(D)
PPAR- α
(PDB ID: 2P54)
Binding Energy
-7.4 kcal/mol
Epicatechin
(E)
PPAR- α
(PDB ID: 2P54)
Binding Energy
-7.7 kcal/mol
Catechin
